# Evidence for Sexual Dimorphism in the Plated Dinosaur *Stegosaurus mjosi* (Ornithischia, Stegosauria) from the Morrison Formation (Upper Jurassic) of Western USA

**DOI:** 10.1371/journal.pone.0123503

**Published:** 2015-04-22

**Authors:** Evan Thomas Saitta

**Affiliations:** School of Earth Sciences, University of Bristol, Bristol, United Kingdom; Scientific Research Centre, Slovenian Academy of Sciences and Arts, SLOVENIA

## Abstract

Conclusive evidence for sexual dimorphism in non-avian dinosaurs has been elusive. Here it is shown that dimorphism in the shape of the dermal plates of *Stegosaurus mjosi* (Upper Jurassic, western USA) does not result from non-sex-related individual, interspecific, or ontogenetic variation and is most likely a sexually dimorphic feature. One morph possessed wide, oval plates 45% larger in surface area than the tall, narrow plates of the other morph. Intermediate morphologies are lacking as principal component analysis supports marked size- and shape-based dimorphism. In contrast, many non-sex-related individual variations are expected to show intermediate morphologies. Taphonomy of a new quarry in Montana (JRDI 5ES Quarry) shows that at least five individuals were buried in a single horizon and were not brought together by water or scavenger transportation. This new site demonstrates co-existence, and possibly suggests sociality, between two morphs that only show dimorphism in their plates. Without evidence for niche partitioning, it is unlikely that the two morphs represent different species. Histology of the new specimens in combination with studies on previous specimens indicates that both morphs occur in fully-grown individuals. Therefore, the dimorphism is not a result of ontogenetic change. Furthermore, the two morphs of plates do not simply come from different positions on the back of a single individual. Plates from all positions on the body can be classified as one of the two morphs, and previously discovered, isolated specimens possess only one morph of plates. Based on the seemingly display-oriented morphology of plates, female mate choice was likely the driving evolutionary mechanism rather than male-male competition. Dinosaur ornamentation possibly served similar functions to the ornamentation of modern species. Comparisons to ornamentation involved in sexual selection of extant species, such as the horns of bovids, may be appropriate in predicting the function of some dinosaur ornamentation.

## Introduction

The genus *Stegosaurus* [1] can be found in the Upper Jurassic Morrison Formation of the western United States, although it has been recovered from Portugal as well [2]. It was an herbivorous quadruped with a small head, long tail, stout forelimbs, and long, columnar hind limbs. *Stegosaurus* had parasagittal dermal armor along its back consisting of vertically oriented plates that varied in size and shape from the neck to the tail and two pairs of long spikes at the end of the tail [3]. Once thought to have had 17 plates, a new specimen has been discovered with 18 [4]. The most common reconstruction puts the plates in two, staggered rows extending along the back of the animal, an idea supported by known articulated specimens [5] and the fact that no two plates on any individual were exactly the same size and shape [6]. Preserved Sharpey’s fibers indicate the orientation of ligaments that held the plates up in a vertical orientation [7]. The tail spikes were arranged in two pairs at the end of the tail, with the larger pair more anterior. The spikes are thought to have exhibited a more posterolateral orientation compared to the plates [5].

Studying sexual selection in dinosaurs poses a challenge because distinguishing sexual dimorphism from non-sex-related individual, interspecific, and ontogenetic variation is difficult [8,9]. Small sample sizes also hinder efforts to statistically demonstrate sexual dimorphism [10]. Previous attempts to identify sexual dimorphism in dinosaurs have not fully considered alternative explanations [11–23], and early work was not quantitative [24,25]. Despite strong evidence for sexual dimorphism in the pterosaurs *Darwinopterus* [26] and *Hamipterus* [27], some researchers have proposed that dinosaur ornamentation, such as the back plates of *Stegosaurus*, was not sexually dimorphic and instead was used for species recognition [28–32] or was under mutual sexual selection [33]. A lack of demonstrated instances of sexual dimorphism in dinosaurs is often cited as evidence for a lack of sexual selection occurring in the group [28–32].

Previous claims of sexual dimorphism within Stegosauria have suffered from the same issues as other attempts to observe sexual dimorphism in non-avian dinosaurs. Geometric morphometrics identified two types of proximal-end femur shape independent from overall size differences in *Kentrosaurus aethiopicus* [22]. The ratio of the occurrence of robust femora to gracile femora was about 2:1. However, slight differences in long bones could be a result of non-sex-related individual or ontogenetic variation. This study did not examine the ontogeny of these femora using histological thin sections. The long bones of dinosaurs grew along their long axis by ossification of cartilage at their proximal and distal ends [34]. As the ossified portions of these femora were the only parts preserved during fossilization, ontogeny might affect the morphology observed at their ends. Other studies found that in some individuals of *K*. *aethiopicus* [15] and *Dacentrurus armatus* [19], which normally have four pairs of sacral ribs, the first sacral vertebra provides an extra set of sacral ribs. Due to the isolated nature of these bones, this variation could not be correlated to any other variation in the skeleton. However, one specimen of *K*. *aethiopicus* has robust femora and four sacral ribs [35,36]. Both sacral types can be observed in different specimens of *Stegosaurus*. Variation in sacral rib count has been interpreted as sexual dimorphism without investigation into possible non-sex-related individual or ontogenetic explanations. Claims of sexual dimorphism in stegosaurs, like those for other non-avian dinosaurs, did not test all alternate hypotheses for the variation they observed, making them inconclusive.

*S*. *mjosi* is the easiest species to diagnose within the genus. It should be noted that while *S*. *mjosi* is used here, this species has been previously referred to as *Hesperosaurus mjosi*, and its taxonomic status is debated [37–39]. Arguments for the validity of *Hesperosaurus* as a separate genus from *Stegosaurus* likely need further addressing. The holotype specimen is HMNS 14 (previously HMNS 001 [37]). Three more specimens have been discovered at the Howe Ranch in Wyoming [4,38,40]. These include SMA 0092 (previously SMA L02 [38]), VFSMA 001 (previously SMA 3074-FV01 [39] and SMA M04 [38]), and SMA 0018 (previously SMA V03 [38]). At least five new individuals are added here from the JRDI 5ES Quarry (S1 Fig) near Grass Range, Montana, along with at least two individuals from the Meilyn Quarry in Como Bluff, Wyoming [41], based on the number of pelves. Among other diagnostic characters (especially in the vertebral series and pelvis [37–39]), the most important diagnostic character of this species is the teardrop-shaped, non-bifurcated tips of neural spines on the anterior caudal vertebrae, making *S*. *mjosi* unique among North American stegosaurs.

The shapes and sizes of forty *S*. *mjosi* plates were examined along with those from three articulated specimens of other species of *Stegosaurus* for comparison. Plate measurements were taken by hand using calipers and a tape measure as well as from scaled photographs. The most likely lateral outline was determined by examining the plate for broken edges, and each fairly complete plate was categorized as either complete enough for an accurate outline to be reconstructed (n = 26 for *S*. *mjosi*) or not entirely complete, but still allowing for a plausible outline to be reconstructed (n = 14 for *S*. *mjosi*). Principal component analysis (PCA) was carried out on the *S*. *mjosi* plates to observe variation in size and shape. In addition to investigating the taphonomy of the JRDI 5ES Quarry, the ontogenetic statuses of the stegosaur bones were examined using the same methodology of previous histological studies of *Stegosaurus* [42–44]. This histological analysis involved X-ray computed tomography (CT) scans of 11 plates and four tail spikes and thin sections of samples taken from the base, middle, and apex of nine plates as well as from the midshaft of a tibia and femur.

## Results

**Fig 1:**
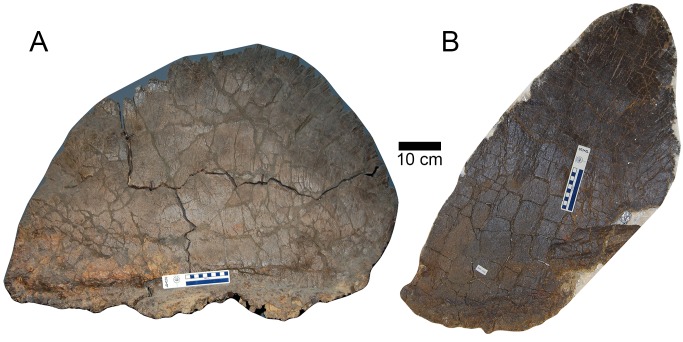
Sexual dimorphism in the plates of *S*. *mjosi*. (A) The largest wide morph plate (SMA 0018). (B) The largest tall morph plate (JRDI 5ES-552).

The sample of plates shows that there are two distinct morphs (S1, S2 Datasets). Plates of one morph are oval and wider than they are tall (Fig 1A). Plates of the other morph are taller than they are wide and sometimes come to a point at their apex (Fig 1B). Wide morph plates reach surface areas 45% greater than do tall morph plates (Fig 1 and S2–S4 Figs). Both morphs of plates can be classified as cervical, dorsal, or caudal based on morphology, size, taphonomy, and comparison to articulated specimens of other species of *Stegosaurus*.

### Principal Component Analysis

**Fig 2:**
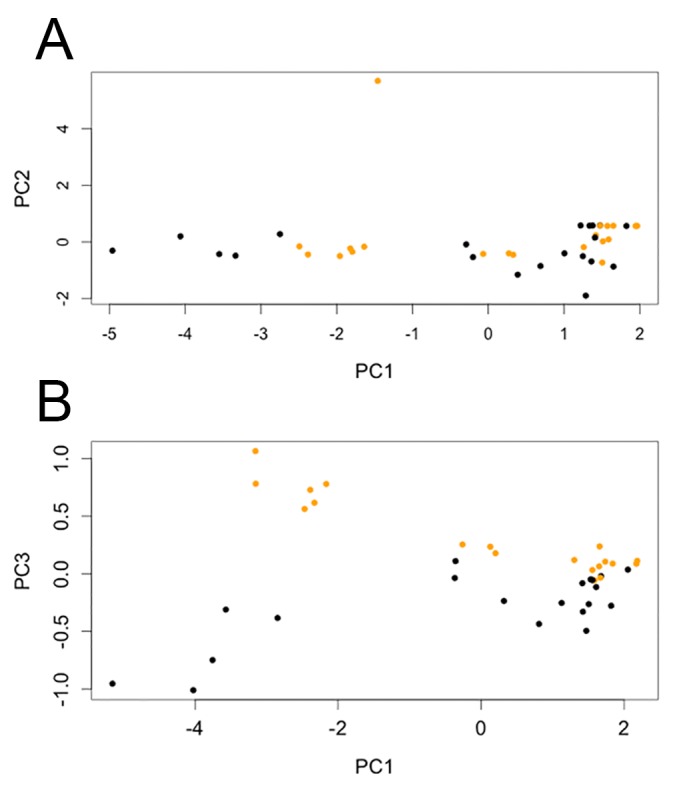
PCA of *S*. *mjosi* plates. (A) The biplot of the first and second principal components for PCA of all fairly complete *S*. *mjosi* plates. The data point with the high PC2 value is the anterior dorsal plate from SMA 0092 that was later dropped from the analysis (n = 40). (B) The biplot of the first and third principal components for PCA of all fairly complete plates except the anterior dorsal plate from SMA 0092 (n = 39). Orange and black points are plates identified to be of the tall and wide morph, respectively.

PCA on six measurements (S3–S6 Datasets) reveals that plates of the two morphs occupy different morphospaces when examined along the first and third principal components (Fig 2B). One plate from SMA 0092 (#1 in S3B Fig) was deemed an outlier and removed from the analysis as it masked the variation in the rest of the sample (Fig 2A). Decreasing values of PC1 indicate larger perimeter, surface area, and base length. With decreasing PC1 values, tall morph plate variation follows a trend of narrowing ‘width’ and increasing distance between base center and apex, while wide morph plate variation follows a trend of increasing ‘width’. ‘Width’ values were taken as either the major or minor axis of the plate as was most appropriate based on that plate’s shape (typically the major axis in wide morphs and minor axis in tall morphs). Divergence in shape is particularly apparent in large plates. This pattern is still observable even when PCA is run on only the most complete plates (S5 Fig). The angle between the base and apex of the plate had a much greater loading on the second principal component than did any of the other variables (S1 Table). Articulated *Stegosaurus* specimens show that this angle decreases posteriorly along the back (S6 Fig). This same trend occurs in plates of *S*. *mjosi*, so both morphs show similar variation along the second principal component.

### Taphonomy of the JRDI 5ES Quarry

All of the stegosaur bones come from a single stratigraphic horizon. The quarry is composed of mudstone. No other macroscopic fossil taxa are found in the bone layer other than stegosaurs, which is unusual for large Morrison Formation dinosaur quarries. A sauropod specimen, whose description is awaiting publication, has been found in a horizon above the stegosaur bed. Carbonate concretions are often found on and around the bones. Small amounts of lignite are occasional found in the quarry, but these are far from common. Evaporites, specifically gypsum roses, are found in abundance throughout the quarry. The long axes of bones do not appear to orient in a common direction (Dean Richmond, personal communication, 2014). Many bones show association and an articulated hindlimb was uncovered, although the degree of association varies throughout the quarry—making precise associations of plates to elements of the body skeleton difficult (S1 Fig). Many small and fragile bones such as unguals and two articulated skulls are present in the quarry. The bone surfaces show no wear or polishing and the bones are not separated into Voorhies groups [45]. There is an apparent lack of bite marks on the stegosaur bones and a lack of shed theropod or crocodilian teeth around them.

There are at least five individuals based on the number of pelves. The different individuals are preserved close together in space, and it seems that the bones of one individual are sometimes intermixed with those of another. Overall, the bones in the quarry have a jumbled pattern.

Of the fairly complete plates found within the bone bed, four can be classified as wide morph and five can be classified as tall morph. Two other plates are not very complete, although one is likely a wide morph (#7 in S2A Fig). Within this quarry and among all other *S*. *mjosi* specimens, regardless of plate morph, all of the other bones of the skeleton come in one variety.

### Histological Analysis

Initial observations of the JRDI 5ES Quarry plates show that many exhibit well-developed, rugose bases with what appear to be large vascular pipes entering through the underside of their bases as well as prominent vasculature across their surface. However, the tall morph plates were observed to generally have more rugose bases as well as deeper, more numerous surface vessels than do the wide morph plates.

**Fig 3:**
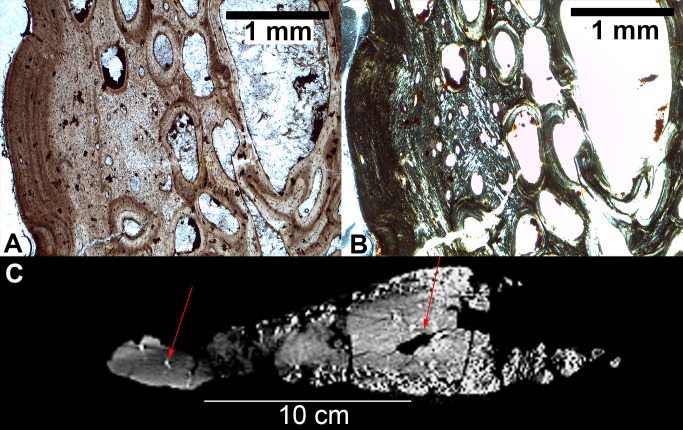
Histology of *S*. *mjosi* plates. (A) A thin section of a tall morph plate (JRDI 5ES-357) in plane polarized light showing an external fundamental system (EFS) indicating the cessation of growth. Bone surface is to the left. (B) The same image but in crossed polarized light. (C) A CT scan of a tall morph plate (JRDI 5ES-237) in cross-section along the frontal plane. Red arrows indicate internal vascular piping that is a sign of sexual maturity.

CT scans and thin sections reveal that all plates found in the quarry show the presence of large, internal vascular piping. They also all show histological features of decreased or ceased growth and extensive remodeling such as laminar/longitudinal channel arrangement, lines of arrested growth (LAGs), and high concentrations of secondary osteons (Fig 3A–3C and S7–S19 Figs and S2–S10 Tables). Some of the internal pipes appear to be clear of infill. Others appear infilled with sediment or with high-density iron. The piping mostly occurs within or near the base (S7 Fig), and a few can be observed to connect to the surface at midplate (S13G Fig). Calcite and iron infilling among the porous spaces of the bone are common as are internal, microscopic fractures of the bones as a result of diagenesis. Some samples from the plates consist almost entirely of secondary bone. The bases of the plates appear histologically ‘younger’ than the middle and apex portions. This pattern has been observed in previous studies of stegosaur plate histology and has been attributed to the process of basal growth in plates [7,44].

The tall morph plates in the quarry tend to have more pronounced internal vascular piping in terms of diameter and number than do the wide morph plates. Tall morph plates also have a larger set of histological markers of maturity than do the wide morph plates, including two large plates with an external fundamental system (EFS) (Fig 3A and 3B and S8 Fig). One of these two plates is the largest tall morph plate in the sample.

Axial channels are present in all of the tail spikes examined (S19 Fig). The femur (S11 Table) and tibia (S12 Table) show features indicating decreased growth rate and increased remodeling such as longitudinal/laminar channel arrangement, LAGs, and high concentrations of secondary osteons (S18 Fig). However, neither appears to have had completely ceased growth nor contain medullary bone.

## Discussion

The dimorphism in the plates could be explained by several alternative hypotheses other than sexual dimorphism: non-sex-related individual variation, one individual possessing both morphs of plates, interspecific variation, and ontogenetic variation.

### Alternate Hypothesis: Non-sex-related Individual Variation

The dimorphism is not a result of non-sex-related individual variation. PCA, combined with simple observation, demonstrates that intermediately shaped plates are lacking. Clearly intermediate morphologies would typically be expected under non-sex-related individual variation. Results such as these satisfy previously proposed criteria for quantitative evidence of size- and shape-based dimorphism [20].

### Alternate Hypothesis: One Individual Possessing Both Morphs of Plates

The two plate morphs do not occur in any single individual. The dimorphism occurs along the entire plates series as both morphs can be classified into cervical, dorsal, or caudal plates. As plates of both morphs demonstrate a range of angle values similar to that seen in the complete, articulated specimens (S20 Fig), both represent plates from all regions of the plate series from head to tail. The size of *S*. *mjosi* plates also peaks at roughly the same angle value as seen on the articulated specimens (~70°). Furthermore, all isolated specimens of *S*. *mjosi* possess plates of solely one morph. SMA 0092 is a tall morph individual while HMNS 14, SMA 0018, and VFSMA 001 are wide morph individuals.

### Alternate Hypothesis: Interspecific Variation

The dimorphism is not a result of interspecific variation. Taphonomy of the JRDI 5ES Quarry suggests that individuals of both morphs co-existed, and possibly comprised a social group, because they were likely together at the time of death and their bodies were not transported before burial. It should be noted that being together at the time of death does not necessarily imply sociality as mass death assemblages of asocial species can be found in the fossil record [46]. The single stratigraphic horizon implies a simultaneous burial for the different individuals. The paleocurrent was weak as indicated by the mudstone lithology as well as the lack of common orientation among the long axes of bones, suggesting a low energy depositional environment. The lack of other macroscopic fossil taxa in the stegosaur bed might have been due to eutrophic or ephemeral conditions. The presence of associated elements as well as small and fragile bones suggests little transportation before burial. As the bones show no polishing or wear and are not divided into Voorhies groups, any large degree of water transportation is unlikely. The lack of bite marks and shed teeth suggests that scavengers did not transport the bones.

The jumbled pattern of bones in the quarry is likely due to disassociation as a result of decomposition of the carcasses prior to burial as well as movements of the bones post-burial as the clays swelled and contracted through hydration and desiccation. Bone disassociation as a result of swelling and contracting clays is seen in other Morrison dinosaur quarries such as the Cleveland-Lloyd Quarry [47].

The group in the JRDI 5ES Quarry appears to be monospecific. If the two morphs represented different species, morphological features that might indicate niche partitioning would be expected, such as in the skull or limbs. Instead, only the plates were found to be dimorphic after examination of the JRDI 5ES Quarry specimens and all other *S*. *mjosi* specimens. Although the largest isolated specimens of *S*. *mjosi* are wide morphs, there is not enough evidence as of yet to determine if the two morphs showed differences in body size. The JRDI 5ES Quarry is the first instance of a multi-individual, monospecific Morrison Formation site to contain *Stegosaurus*.

While both tall and wide morphs plates have been found in the Meilyn Quarry, the lithology suggests fluvial transportation and other dinosaur species are found alongside the stegosaur individuals [41].

### Alternate Hypothesis: Ontogenetic Variation

The dimorphism is not a result of ontogenetic variation. Prior histological work has already provided some evidence against this alternate hypothesis. The wide morph is known from fully-grown, old adults specimens HMNS 14 [44] and SMA 0018 [43]. Wide morph specimen VFSMA 0001 and tall morph specimen SMA 0092 were sexually mature adults that were still growing [43].

The JRDI 5ES Quarry bone bed has produced a pair of femora only 70 cm in length (compared to the maximum size of *S*. *mjosi* femora at 96 cm) as well as the largest known tall morph plate. As the individuals from this quarry come in a range of sizes, the possibility of ontogenetic variation had to be examined. Initial observations hinted at the presence of maturity in the quarry, as there are very large plates of both morphs that often show well-developed external vasculature.

Both morphs of plates in the quarry show indicators of sexual maturity described by previous researchers [42–44]. Internal, vascular piping in the plates is one such indication. Histological features further show reduced or halted growth and increased remodeling that appear after sexual maturity and as the individual reaches full size. The presence of an EFS in two of the tall morph plates indicates that these plates had ceased their growth and came from fully-grown adults.

The wide morph plates tend to show features of young adults that are sexually mature, yet still growing, while the tall morph plates tend to show more features of fully-grown, old adults. The slight difference in ontogeny between the two morphs in the quarry is further evidence that the two morphs of plates come from separate individuals rather than from different positions along the back of one animal. However, it cannot be said with confidence how many individuals of each morph contributed to the collection of plates recovered at the JRDI 5ES Quarry. All that is known is that there are at least five individuals found in the quarry based on the number of pelves, and that the plates from this site must come from at least two individuals, one of each morph.

The axial channels in the spikes are evidence of the presence of old adults [44] while the histology of the tibia and femur are evidence of the presence of sexually mature adults that are still growing [43].

In combination with previous histological studies on other specimens, plates of both morphs of *S*. *mjosi* came from sexually mature, young adults as well as fully-grown, old adults. The discovery of an old adult, tall morph individual that was fully-grown completes the expected ontogenetic ranges seen in both morphs under the premise of sexual dimorphism. One morph is not the immature form of the other.

## Conclusions

With all alternate hypotheses apparently ruled out, sexual dimorphism is the most likely explanation for the observed variation in the plates. The evidence provided here is the first support for sexual dimorphism in a non-avian dinosaur that rules out all other possible explanations for the observed morphological variation. More importantly, the dimorphism occurs in the ornamentation. As a result, *S*. *mjosi* ornamentation was likely a secondary sexual characteristic.

No medullary bone was found in the femur or tibia sampled from the JRDI 5ES Quarry that might allow for one of the morphs to be assigned as definitively female. Without this particular tissue, and without any specimens preserved with eggs inside the body cavity, it is not possible to assign sexes to the two morphs with absolute certainty. However, modern analogs might be able to provide clues.

**Fig 4:**
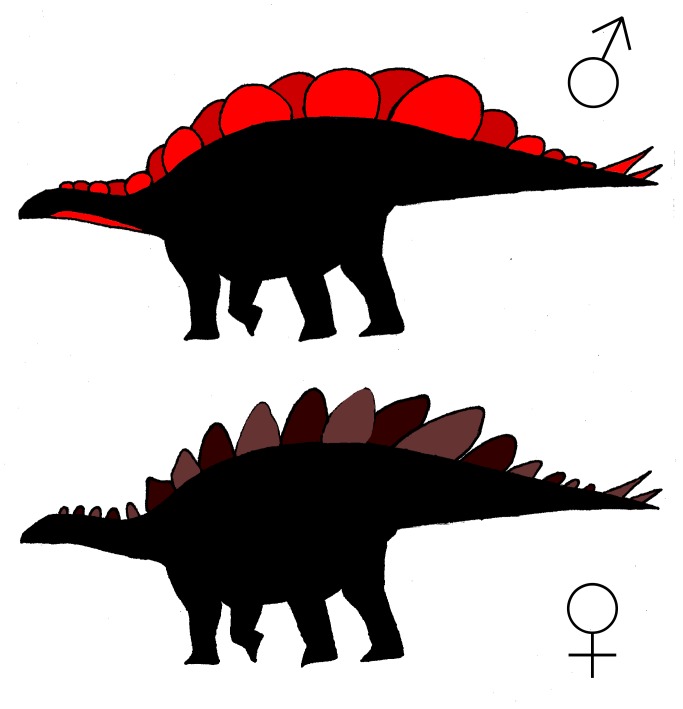
Hypothetical silhouettes of male and female *S*. *mjosi*. The wide morph exhibits more overlap between adjacent plates than does the tall morph, leading to a more continuous display surface. Sexual dimorphism in the size and shape and plates might have also occurred with other sexual differences such as sexual dichromatism.

Stegosaurs were very unusual animals, and no extant species are perfect analogs. However, bovids might be one of the best options. In addition to both being large, quadrupedal herbivores, stegosaur plates and modern bovid horns are both composed of a boney core surrounded by a keratin sheath. Applying the same reasoning garnered from studies of modern bovid horns [48–50] to *S*. *mjosi* plates, the wide morph may represent the male while the tall morph could represent the female (Fig 4). Compared to females, males are typically expected to invest more energy into growing and maintaining their ornamentation. Wide morph plates are 45% larger in maximum surface area than tall morph plates, and an energetics perspective would assign the wide morph as male. The larger wide morph plates were probably under sexual selection like male bovid horns and functioned to create a broad, continuous display surface along the animal’s back, like a billboard. Unlike sparring male bovids, the nature of *Stegosaurus* plates suggests that sexual selection occurred through female mate choice rather than male-male competition. In contrast to the immobile, vertical plates, tail spikes might have been able to function in antagonistic behavior. However, male-male competition is unlikely due to the possible lethality of *Stegosaurus* spikes [5,51,52]. In contrast, the ornaments of male bovids are typically thought to have evolved for non-lethal confrontation [48,49]. Furthermore, if *Stegosaurus* used its spikes in male-male competition, dimorphism would be expected in the spikes rather than the plates. The tall morphs, due to their more erect angle and pointed apex, were probably under natural selection like female bovid horns and functioned as prickly predator deterrents.

The function of *Stegosaurus* plates, along with the ornamentation of other dinosaur species, has been a long-running debate in paleontology. These results suggest that *Stegosaurus* plates possibly had multiple functions because, despite the presence of sexual dimorphism, females still possessed plates. Based on an understanding of modern species, these likely included sex-related display and defense.

These results may suggest that previously described ornament variations in dinosaurs are actually cases of sexual dimorphism. For example, the ontogeny of *Triceratops* horns [53] seems to match sexual variation seen in modern bovid horns, where males have horns with downward pointing tips that are better for sparring and resistance of lateral stresses and females have thinner and straighter horns with tips that point up and away from the skull that are more efficient stabbing weapons [48]. With the first evidence of sexual dimorphism in a dinosaur species, future histological research could examine the possibility of some *Triceratops* specimens with “juvenile” horns being sexually mature and fully-grown.

## Materials and Methods

The plate morphologies of all available *S*. *mjosi* specimens identified by this study were examined (HMNS 14, SMA 0018, SMA 0092, VFSMA 001, as well as newly identified specimens housed at the Wyoming Dinosaur Center and Judith River Dinosaur Institute). Additionally, several articulated *Stegosaurus* specimens not of the species *S*. *mjosi* (DMNS 2818, NHMUK R36730, and USNM 4934) were examined for comparison. NHMUK R36730 has been previously referred to as SMA DS-RCR-2003-02 [54], SMA RCR0603 [43], and SMA S01 [38]. Specimens used in this study are housed in the Denver Museum of Nature and Science (DMNS), Hayashibara Museum of Natural Sciences (HMNS), Judith River Dinosaur Institute (JRDI), Natural History Museum in London (NHMUK), Sauriermuseum Aathal (SMA), United States National Museum (USNM), Verein für das Sauriermuseum Aathal (VFSMA), and Wyoming Dinosaur Center (WDC). The CT scans, in CD-ROM format, and the histological thin section slides of the JRDI 5ES Quarry specimens are housed with the specimens themselves at the Judith River Dinosaur Institute.

After establishing a quantitative basis for dimorphism in plate shape, this variation was tested against various alternate explanatory hypotheses. This involved further methodologies including examination of the taphonomy of the JRDI 5ES Quarry using the quarry map and basic sedimentology of the site, X-ray computed tomography (CT) scans of the JRDI 5ES plates and spikes, and the preparation of histological thin sections of samples taken from JRDI 5ES plates and long bones.

### Plate Measurements

**Fig 5:**
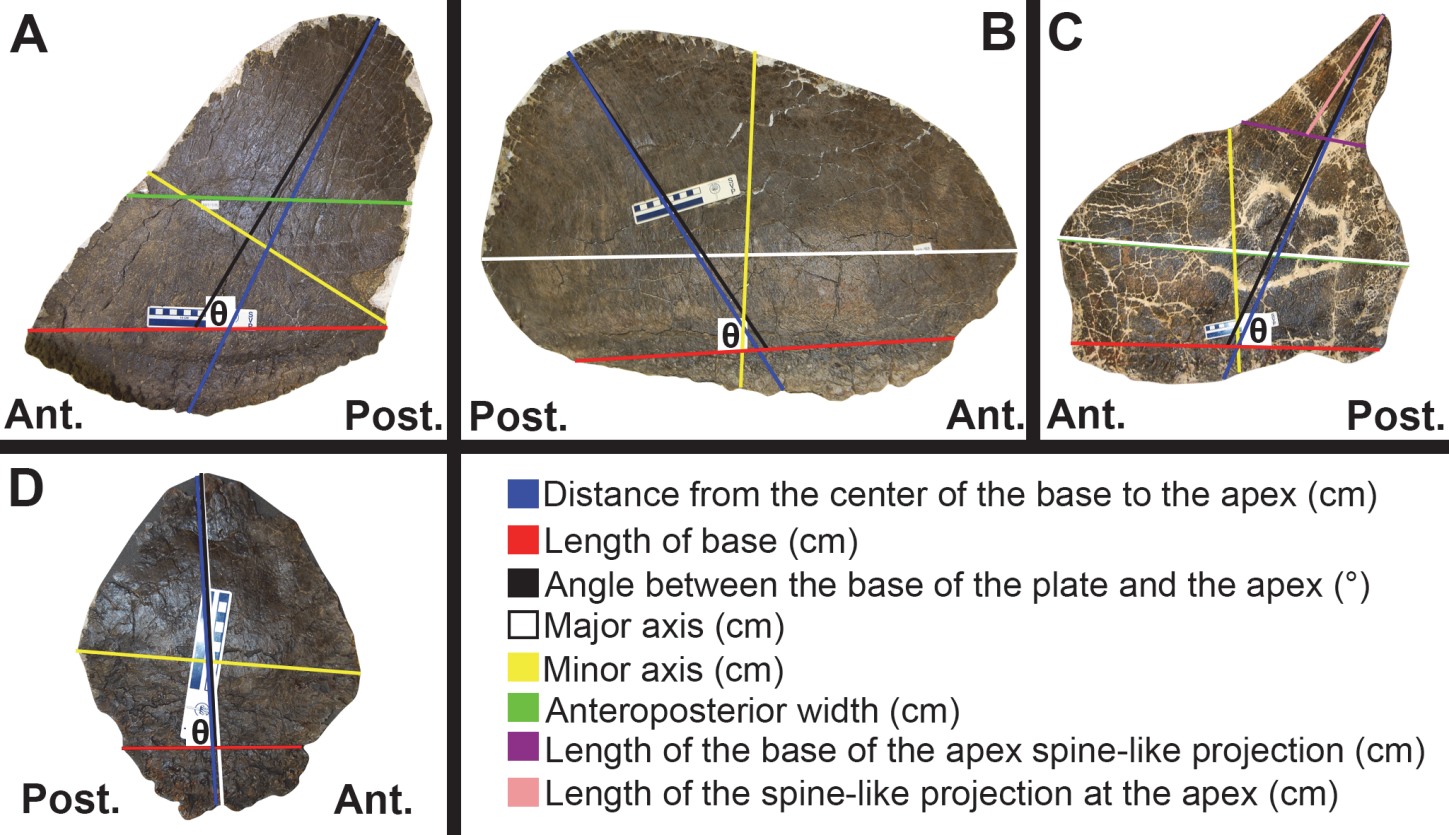
Diagrammatic depiction of measurements. (A) Vertical oval/triangular plates (JRDI 5ES-579). Tall morph *S*. *mjosi* plates typically fall under this category. The minor axis, rather than the anteroposterior width, was the value used for ‘width’ in PCA. (B) Horizontal oval plates (JRDI 5ES-523). Wide morph *S*. *mjosi* plates typically fall under this category. The major axis was the value used for ‘width’ in PCA. (C) Sub-right trapezoidal plates (DMNS 2818). The major axis and anteroposterior width tend to converge. The major axis was the value used for ‘width’ in PCA. (D) Shoulder plates (NHMUK R36730). These plates have a distinctive diamond shape. The distance from the center of the base to the apex and the major axis tend to converge.

Measurements were made by hand and from scaled photographs taken with a Nikon D40 camera (18–55 mm lens) using Adobe Photoshop CC on a MacBook 7 with 2 GB of RAM and a 2.4 GHz Intel Core 2 Duo processor. As some plates were on mounted specimens and were difficult to get close to, the distance at which photographs were taken varied. Photographs were taken at a distance as close to the plate as possible such that the entire plate was included in the frame and the plate was viewed in lateral profile. Measurements for PCA were made of the angle between the center of the base and the apex in degrees, the length of the base in cm, the perimeter of the plate in cm, the ‘width’ of the plate in cm (typically the major axis in wide morphs and minor axis in tall morphs), the distance from the center of the base to the apex in cm, and the surface area of the plate in cm^2^ (Fig 5). The angle between the base of the plate and the apex was taken as the acute angle formed between the line connecting the anterior and posterior limits of the base and the line connecting the apex to the midpoint of the previous line. The shape of the large caudal plate on wide morph specimen HMNS 14 (#1 in S4A Fig) lent itself to have ‘width’ measured in the same manner as those of tall morph plates such that ‘width’ equals the minor axis. The shape of the first dorsal plate on tall morph specimen SMA 0092 (#1 in S3B Fig) lent itself to have ‘width’ measured in the same manner as those of wide morph plates such that the ‘width’ equals the major axis. Surface area was calculated to the nearest cm^2^ by measuring the area of one side of a plate from a scaled photograph, and then multiplying by two to get the whole surface area of the plate. The base of the plate, presumably embedded in the skin during life, was included in this measure since it is difficult to estimate what portion of a plate was exposed above the skin. A plausible lateral outline for each plate was determined to the highest accuracy that the completeness of the fossil allowed. To determine this, plates were examined for broken edges and thickness measurements were taken at various points along the edge using a pair of digital calipers. If a plate is unusually thick at one location, then it is likely that this edge is broken because most plates continuously thin from base to apex and from the center to the edges. The same outline used to calculate surface area was used to determine the perimeter of the plate. Principal component analysis was done using the prcomp() function of stats package version 3.1.0 in R version 0.98.501 (S1 Script). Default options were used for prcomp() except that scale = TRUE. S6 Fig and S20 Fig were also created in R.

### Histological Analysis

CT scanning was done at the radiology department of Billings Clinic in Billings, Montana (Siemens Somatom Sensation 64, Siemens corporation, 0.6 mm acquisition, 1.0 mm slice thickness, 140 kV, 180 mA). Eleven plates (JRDI 5ES-237, JRDI 5ES-256, JRDI 5ES-296, JRDI 5ES-357, JRDI 5ES-401, JRDI 5ES-518, JRDI 5ES-523, JRDI 5ES-525, JRDI 5ES-552, JRDI 5ES-553, JRDI 5ES-579), one of which was only a fragment of a base, and four tail spikes (JRDI 5ES-232, JRDI 5ES-245, JRDI 5ES-258, JRDI 5ES-260) were scanned. Scans were saved onto CD-ROM and then viewed using Syngo FastView software on a Dell desktop with 32 GB of RAM and a 3.6 GHz Intel Core i7-3820 processor

Samples for thin sectioning were taken from the base, middle, and apex of nine plates from the JRDI 5ES Quarry as well as from midshaft on the cranial surface of one femur (JRDI 5ES-229) and one tibia (JRDI 5ES-501) along the mediolateral plane. Wedge-shaped samples approximately 2 cm wide and 2 cm tall were cut from the plates and tibia using a dremel saw with a diamond-coated blade. The sample from the femur was taken using a drill press to retrieve a core approximately 1 cm in diameter from midshaft. These samples were then sent to Spectrum Petrographics, Inc. in Vancouver, Washington to be made into 27 mm by 46 mm slides embedded in EPOTEK 301 and cut to 30 microns in thickness. Slides were analyzed using a Leica DM750 light microscope with plane and crossed polarized light settings and equipped with a Leica ICC50 HD camera. These images were then viewed using Leica Acquire software.

### Plate Numbering of Articulated Specimens

The following reasoning was applied in order to determine plate numbers on articulated *Stegosaurus* specimens for S6 Fig USNM 4934 has 17 plates [39,55], the anterior 11 of which are inaccessible due to the current position of the specimen in its display. It should be noted that previous reconstructions have also combined material from USNM 4934 and USNM 4714 [6], with some even adding an 18^th^ plate [56]. DMNS 2818 has 16 plates [5,57,58]. NHMUK R36730 has 18 plates, more than any other stegosaur previously discovered [4]. Therefore, it is likely that all *Stegosaurus* individuals had 18 plates and it was assumed that specimens with fewer plates are incomplete rather than representing biological variation in plate number. The degree of articulation of USNM 4934 decreases from anterior to posterior along the specimen. It seems probable that USNM 4934 is missing its most posterior plate (plate #18) and that DMNS 2818 is missing its last two most posterior plates (plates #17 and #18). These posterior tail plates would be smaller than the tail plates that were preserved with the specimens, making them more likely to be transported away from the rest of the body. As for NHMUK R36730, the method of numbering the plates follows that used in the mounted cast at the Sauriermuseum Aathal [4] with three modifications (S21 Fig). First, in the mounted specimen, a gap in the series of plates was inferred and filled in with a hypothetical 19^th^ plate (placed in the 14^th^ plate position on the mount). There is no reason to add an extra plate when only 18 were discovered, so this hypothetical plate was ignored. Second, the plate originally described as the 5^th^ plate is now plate #18 because its morphology clearly matches those of distal tail plates in other *Stegosaurus* specimens. Third, the plate originally described as the 15^th^ plate is now plate #11 because this plate was found isolated from the rest of the body close to the anterior half of the skeleton. It was assigned the 11^th^ position because it has a surface area in-between the plates directly anterior and posterior to it. By labeling this plate as a dorsal plate rather than a caudal plate, it results in plate #13 being the largest on the body. The largest plate on DMNS 2818 is also plate #13 [5,58].

## Supporting Information

S1 Dataset Raw measurements of plates in a. xlsx format. (XLSX)

S2 Dataset RData file containing the data to run S1 Script. (ZIP)

S3 Dataset csv file containing the PC values for each specimen after a PCA on all *S*. *mjosi* plates under S1 Script. (CSV)

S4 Dataset csv file containing the PC values for each specimen after a PCA on all *S*. *mjosi* plates except the anterior dorsal plate from SMA 0092 under S1 Script. (CSV)

S5 Dataset csv file containing the PC values for each specimen after a PCA on only the most complete *S*. *mjosi* plates under S1 Script. (CSV)

S6 Dataset csv file containing the PC values for each specimen after a PCA on only the most complete *S*. *mjosi* plates except the anterior dorsal plate from SMA 0092 under S1 Script. (CSV)

S1 Fig Enlarged southern portion of the JRDI 5ES Quarry as of September 1, 2013. Direction of modern north is indicated. Fairly complete plates are indicated in blue. As fieldwork has continued in the quarry, a full map has yet to be completed. Therefore, some bones in the northern portion of the quarry have not yet been included. Courtesy of the Judith River Dinosaur Institute.
(TIF)

S2 Fig *S*. *mjosi* cervical plates. (A) Wide morph cervical plates. (1–4) HMNS 14. (5) WDC DMQ-001; J9979. (6) VFSMA 001. (7) JRDI 5ES-553. Images of HMNS 14 courtesy of K. Carpenter. (B) Tall morph cervical plates. (1–7) SMA 0092. (8) WDC DMQ-001; 9791. Plates are hypothetically ordered from anterior-most to posterior-most, although the numbers are not meant to indicate precise plate position and certain plate positions are probably duplicated in the sample. Some images are flipped so the anterior edge is to the left. Color of scale bar (= 10cm) indicates level of completeness. Green—Plates that are complete enough for an accurate outline to be reconstructed. Yellow—Plates that are not entirely complete, but allow for a plausible outline to be reconstructed. Red—Plates that are incomplete and from which an outline cannot be reconstructed.
(TIF)

S3 Fig *S*. *mjosi* dorsal plates. (A) Wide morph dorsal plates. (1) Cast of VFSMA 001. (2) HMNS 14. (3) JRDI 5ES-523. (4) VFSMA 001. (5) HMNS 14. (6) JRDI 5ES-518. (7) JRDI 5ES-256. (8) SMA 0018. Images of HMNS 14 courtesy of K. Carpenter. (B) Tall morph dorsal plates. (1) SMA 0092 that was found to be an outlier in PCA. (2) WDC DMQ-001; from block 9999. (3) JRDI 5ES-237. (4) JRDI 5ES-357. (5) SMA 0092. (6) JRDI 5ES-552. Plates are hypothetically ordered from anterior-most to posterior-most, although the numbers are not meant to indicate precise plate position and certain plate positions are probably duplicated in the sample. Some images are flipped so the anterior edge is to the left. Color of scale bars (= 10 cm) as in S2 Fig.
(TIF)

S4 Fig *S*. *mjosi* caudal plates. (A) Wide morph caudal plates. (1) HMNS 14. (2) JRDI 5ES-525. (3) HMNS 14. (4, 5) VFSMA 001. (6) SMA 0018. (7) VFSMA 001. (8) HMNS 14. Images of HMNS 14 courtesy of K. Carpenter. (B) Tall morph caudal plates. (1) JRDI 5ES-579. (2) JRDI 5ES-401. (3) SMA 0092. (4) WDC DMQ-001; 9808. (5) SMA 0092. (6) WDC DMQ-001; 570P. Plates are hypothetically ordered from anterior-most to posterior-most, although the numbers are not meant to indicate precise plate position and certain plate positions are probably duplicated in the sample. Some images are flipped so the anterior edge is to the left. Color of scale bars (= 10 cm) as in S2 Fig.
(TIF)

S5 Fig Biplot of the first and third principal components for PCA of only the most complete *S*. *mjosi* plates except for the anterior dorsal plate from SMA 0092. Orange and black points are plates identified to be of the tall and wide morph, respectively. Decreasing values of PC1 indicate larger perimeter, surface area, and base length. With decreasing PC1 values, tall morph plate variation follows a trend of narrowing ‘width’ and increasing distance between base center and apex, while wide morph plate variation follows a trend of increasing ‘width’ (n = 25).
(TIF)

S6 Fig Angle between the apex and base (degrees) vs. plate number. The data for articulated specimens of *Stegosaurus*: NHMUK R36730, DMNS 2818, and USNM 4934. Plate numbers start at the anterior of the specimen and increase posteriorly. Highly incomplete plates or plates that are entirely missing from the specimen are not included in the plot (n = 33).
(TIF)

S7 Fig CT scan of a wide morph plate (JRDI 5ES-525) in cross-section along the sagittal plane. Red arrows indicate internal vascular piping.
(TIF)

S8 Fig Bone tissue from midplate on a tall morph plate (JRDI 5ES-552). (A) Under plane polarized light. (B) Under crossed polarized light. Bone surface is to the left. Note presence of EFS.
(TIF)

S9 Fig Bone tissue of wide morph plate JRDI 5ES-256. (A, B) Apex cortical bone under plane polarized light. Midplate cortical (C) and cancellous (D) bone under plane polarized light. Base cancellous (E) and cortical (F) bone under crossed polarized light (Scale bars = 1 mm). Bone surface is towards scale bar in A-C, F. CT cross sections along the transverse (G) and frontal (H) planes. Red arrows indicate internal vascular piping (Scale bars = 10 cm).
(TIF)

S10 Fig Bone tissue of wide morph plate JRDI 5ES-518. The same image of apex cortical bone under (A) plane polarized and (B) crossed polarized light. The same image of midplate cortical bone under (C) plane polarized and (D) crossed polarized light. Base cortical bone under (E) plane polarized and (F) crossed polarized light (Scale bars = 1 mm). Bone surface is towards scale bar in A-F. (G, H) CT cross sections along the transverse plane. Red arrows indicate internal vascular piping (Scale bars = 10 cm).
(TIF)

S11 Fig Bone tissue of wide morph plate JRDI 5ES-523. Apex cortical (A) and cancellous (B) bone under plane polarized light. The same image of midplate cortical bone under (C) plane polarized and (D) crossed polarized light. Base cortical (E) and cancellous (F) bone under plane polarized light (Scale bars = 1 mm). Bone surface is towards scale bar in A, C-E. CT cross sections along the transverse (G) and frontal (H) plane. Red arrows indicate internal vascular piping (Scale bars = 10 cm).
(TIF)

S12 Fig Bone tissue of wide morph plate JRDI 5ES-525. Apex cortical (A) and cancellous (B) bone under plane polarized light. The same image of midplate cortical bone under (C) plane polarized and (D) crossed polarized light. Base cortical (E) and cancellous (F) bone under plane polarized and crossed polarized light, respectively (Scale bars = 1 mm). Bone surface is towards scale bar in A, C-E. (G, H) CT cross sections along the frontal plane. Red arrows indicate internal vascular piping (Scale bars = 10 cm).
(TIF)

S13 Fig Bone tissue of tall morph plate JRDI 5ES-237. The same image of apex cortical bone under (A) plane polarized and (B) crossed polarized light. The same image of midplate cortical bone under (C) plane polarized and (D) crossed polarized light. (E) Base cortical bone under crossed polarized light (Scale bars = 1 mm). Bone surface is towards scale bar in A-E. CT cross sections along the (F) sagittal, (G) frontal, and (H) transverse plane. Red arrows indicate internal vascular piping. Large red arrow in G indicates pipe exiting onto surface of the plate (Scale bars = 10 cm).
(TIF)

S14 Fig Bone tissue of tall morph plate JRDI 5ES-357. The same image of midplate cortical bone under (A) plane polarized and (B) crossed polarized light. The same image of midplate cancellous bone under (C) plane polarized and (D) crossed polarized light. Base cortical (E) and cancellous (F) bone under plane polarized light (Scale bars = 1 mm). Bone surface is towards scale bar in A, B, E. (G, H) CT cross sections along the transverse plane. Red arrows indicate internal vascular piping (Scale bars = 10 cm).
(TIF)

S15 Fig Bone tissue of tall morph plate JRDI 5ES-401. The same image of apex cortical bone under (A) plane polarized and (B) crossed polarized light. (C) Apex cortical bone under plane polarized light. Midplate cancellous (D) and cortical (E) bone under plane polarized light. Base cortical (F) bone under crossed polarized light (Scale bars = 1 mm). Bone surface is towards scale bar in A-C, E, F. CT cross sections along the transverse (G) and frontal (H) plane. Red arrows indicate internal vascular piping (Scale bars = 10 cm).
(TIF)

S16 Fig Bone tissue of tall morph plate JRDI 5ES-552. The same image of apex cortical bone under (A) plane polarized and (B) crossed polarized light. (C) Apex cortical bone under plane polarized light. The same image of base cortical bone under (D) plane polarized and (E) crossed polarized light (Scale bars = 1 mm). Bone surface is towards scale bar in A-C, but are opposite to the scale bar in D, E. CT cross sections along the sagittal (F), transverse (G), and frontal (H) plane. Red arrows indicate internal vascular piping (Scale bars = 10 cm).
(TIF)

S17 Fig Bone tissue of tall morph plate JRDI 5ES-579. The same image of apex cortical bone under (A) plane polarized and (B) crossed polarized light. Apex (C, D), midplate (E), and base (F) cortical bone under plane polarized light (Scale bars = 1 mm). Bone surface is towards scale bar in A-D, F but is opposite to the scale bar in E. CT cross sections along the frontal (G) and transverse (H) plane. Red arrows indicate internal vascular piping (Scale bars = 10 cm).
(TIF)

S18 Fig Bone tissue of JRDI 5ES Quarry limb bones. The femur JRDI 5ES-229 histology from the outer (A), middle (B), and inner (C) regions under crossed polarized light. The tibia JRDI 5ES-501 histology from the outer (D), middle (E), and inner (F) regions. D is under plane polarized light while D, F are under crossed polarized light (Scale bars = 1 mm). Periosteal surface is towards the top and endosteal surface is towards the bottom in all images.
(TIF)

S19 Fig CT scans of JRDI 5ES Quarry tail spikes. Cross sections of posterior spikes (A) JRDI 5ES-245 and (B) JRDI 5ES-258 along the sagittal and frontal plane, respectively. Cross sections of anterior spikes (C) JRDI 5ES-232 and (D) JRDI 5ES-260 along the frontal and sagittal plane, respectively. Red arrows indicate axial channel (Scale bars = 10 cm).
(TIF)

S20 Fig Angle between the apex and base (degrees) vs. plate surface area (cm^2^). (A) The data from three articulated specimens of *Stegosaurus*: NHMUK R36730, DMNS 2818, and USNM 4934 (n = 33). (B) The data from the *S*. *mjosi* examined in this study. Orange and black points are plates identified to be of the tall and wide morph, respectively (n = 40).
(TIF)

S21 Fig Corrected plate arrangement on NHMUK R36730. The 18 plates are shown here in correct arrangement from anterior to posterior with the tail spikes shown. The original plate numbers as they were mounted at the Sauriermuseum Aathal are shown below each plate. The color of the number indicates the level of completeness as in S2 Fig The asterisks denote plates that have been rotated by 45° or more and/or flipped in order to properly orient the base of the plate ventrally. Modified from Siber and Möckli [4].
(TIF)

S1 Script R script for PCA, S6 Fig and S20 Fig. (R)

S1 Table PCA variable loadings. PCA 1: Loadings of each variable on the first three principal components for a PCA of all fairly complete *S*. *mjosi* plates. PC1 explains about 63% of the variation. PC1 and PC2 explain about 83% of the variation. PC1, PC2, and PC3 explain about 98% of the variation (n = 40). PCA 2: Loadings of each variable on the first three principal components for a PCA of all fairly complete *S*. *mjosi* plates except the anterior dorsal plate from SMA 0092. PC1 explains about 78% of the variation. PC1 and PC2 explain about 95% of the variation. PC1, PC2, and PC3 explain about 99% of the variation (n = 39). PCA 3: Loadings of each variable for the first three principal components for a PCA of only the most complete *S*. *mjosi* plates except the anterior dorsal plate from SMA 0092. PC1 explains about 79% of the variation. PC1 and PC2 explain about 95% of the variation. PC1, PC2, and PC3 explain about 99% of the variation (n = 25). Table corresponds to biplots in Fig 2 and S5 Fig.
(DOCX)

S2 Table Histological observations from the base, midplate, and apex of wide morph plate JRDI 5ES-256. Histological stage according to Hayashi et al. [42] and ontogenetic status according to Hayashi et al. [44] listed at the bottom. LAG—Line of arrested growth.
(DOCX)

S3 Table Histological observations from the base, midplate, and apex of wide morph plate JRDI 5ES-518. Histological stage according to Hayashi et al. [42] and ontogenetic status according to Hayashi et al. [44] listed at the bottom. LAG—Line of arrested growth.
(DOCX)

S4 Table Histological observations from the base, midplate, and apex of wide morph plate JRDI 5ES-523. Histological stage according to Hayashi et al. [42] and ontogenetic status according to Hayashi et al. [44] listed at the bottom. LAG—Line of arrested growth.
(DOCX)

S5 Table Histological observations from the base, midplate, and apex of wide morph plate JRDI 5ES-525. Histological stage according to Hayashi et al. [42] and ontogenetic status according to Hayashi et al. [44] listed at the bottom. LAG—Line of arrested growth.
(DOCX)

S6 Table Histological observations from the base, midplate, and apex of tall morph plate JRDI 5ES-237. Histological stage according to Hayashi et al. [42] and ontogenetic status according to Hayashi et al. [44] listed at the bottom. LAG—Line of arrested growth.
(DOCX)

S7 Table Histological observations from the base, midplate, and apex of tall morph plate JRDI 5ES-357. Histological stage according to Hayashi et al. [42] and ontogenetic status according to Hayashi et al. [44] listed at the bottom. LAG—line of arrested growth. EFS—External fundamental system.
(DOCX)

S8 Table Histological observations from the base, midplate, and apex of tall morph plate JRDI 5ES-401. Histological stage according to Hayashi et al. [42] and ontogenetic status according to Hayashi et al. [44] listed at the bottom.
(DOCX)

S9 Table Histological observations from the base, midplate, and apex of tall morph plate JRDI 5ES-552. Histological stage according to Hayashi et al. [42] and ontogenetic status according to Hayashi et al. [44] listed at the bottom. LAG—Line of arrested growth. EFS—External fundamental system.
(DOCX)

S10 Table Histological observations from the base, midplate, and apex of tall morph plate JRDI 5ES-579. Histological stage according to Hayashi et al. [42] and ontogenetic status according to Hayashi et al. [44] listed at the bottom. LAG—Line of arrested growth.
(DOCX)

S11 Table Histological observations from the exterior, middle, and interior of the femur JRDI 5ES-229. Histological stage according to Hayashi et al. [42] and ontogenetic status according to Redelstorff & Sander [43] listed at the bottom. No medullary bone was present. LAG—Line of arrested growth.
(DOCX)

S12 Table Histological observations from the tibia JRDI 5ES-501. Histological stage according to Hayashi et al. [42] and ontogenetic status according to Redelstorff & Sander [43] listed at the bottom. No medullary bone was present. LAG—Line of arrested growth. ICL—Inner circumferential layer.
(DOCX)

## References

[1] Marsh OC. A new order of extinct Reptilia (Stegosauria) from the Jurassic of the Rocky Mountains. Am J Sci Series 3. 1877;14: 513–514.

[2] Escaso F, Ortega F, Dantas P, Malafaia E, Pimentel NL, Pereda-Suberbiola X, et al New evidence of shared dinosaur across Upper Jurassic proto-North Atlantic: *Stegosaurus* from Portugal. Naturwissenschaften. 2007;94: 367–374. 17187254 10.1007/s00114-006-0209-8

[3] Galton PM, Upchurch P. Stegosauria In: Weishampel DB, Dodson P, Osmolska H, editors. The Dinosauria. Berkeley: Berkeley University Press; 2004 pp. 343–362.

[4] Siber HJ, Möckli U. The Stegosaurs of the Sauriermuseum Aathal. Aathal, Switzerland: Sauriermuseum Aathal; 2009.

[5] Carpenter K. Armor of *Stegosaurus stenops*, and the taphonomic history of a new specimen from Garden Park, Colorado. Modern Geology. 1998;23: 127–144.

[6] Czerkas SA. A reevaluation of the plate arrangement on *Stegosaurus stenops* In: Czerkas SJ, Olson EC, editors. Dinosaurs Past & Present, Volume 2 Seattle: University of Washington Press; 1987 pp. 82–99.

[7] Buffrénil V de, Farlow JO, Ricqlès A de. Growth and function of *Stegosaurus* plates: evidence from bone histology. Paleobiology. 1986;12: 459–473.

[8] Brusatte SL. Dinosaur Paleobiology, Volume 2 Hoboken: Wiley-Blackwell; 2012.

[9] Knell RJ, Naish D, Tomkins JL, Hone DW. Sexual selection in prehistoric animals: detection and implications. Trends Ecol Evol. 2013;28 1: 38–47. 10.1016/j.tree.2012.07.015 22954658

[10] Prieto-Marquez A, Gignac PM, Joshi S. Neontological evaluation of pelvic skeletal attributes purported to reflect sex in extinct non-avian archosaurs. J Vert Paleontol. 2007;27: 603–609.

[11] Galton PM. The ornithischian dinosaur *Hypsilophodon* from the Wealden of the Isle of Wight. Bull Brit Mus (Nat Hist) Geol. 1974;25: 1–152c.

[12] Dodson P. Taxonomic implications of relative growth in lambeosaurine hadrosaurs. Syst Biol. 1975;24: 37–54.

[13] Dodson P. Quantitative aspects of relative growth and sexual dimorphism in *Protoceratops* . J Paleontol. 1976;50: 929–940.

[14] Chapman RE, Galton PM, Sepkoski JJ Jr, Wall WP. A morphometric study of the cranium of the pachycephalosaurid dinosaur *Stegoceras* . J Paleontol. 1981;55: 608–618.

[15] Galton PM. The postcranial anatomy of stegosaurian dinosaur *Kentrosaurus* from the Upper Jurassic of Tanzania, East Africa. Geologica et Palaeontologica 1982;15: 139–160.

[16] Chapman RE. Shape analysis in the study of dinosaur morphology In: Carpenter K, Currie PJ, editors. Dinosaur Systematics: Approaches and Perspectives. Cambridge: Cambridge University Press; 1990 pp. 21–42.

[17] Lehman TM. The ceratopsian subfamily Chasmosaurinae: sexual dimorphism and systematics In: Carpenter K, Currie PJ, editors. Dinosaur Systematics: Approaches and Perspectives. Cambridge: Cambridge University Press; 1990 pp. 211–229.

[18] Raath MA. Morphological variation in small theropods and its meaning in systematics: evidence from *Syntarsus* In: Carpenter K, Currie PJ, editors. Dinosaur Systematics: Approaches and Perspectives. Cambridge: Cambridge University Press; 1990 pp. 91–105.

[19] Galton PM. Postcranial remains of the stegosaurian dinosaur *Dacentrurus* from the Upper Jurassic of France and Portugal. Geol Palaeont. 21991;5: 299–327.

[20] Chapman RE, Weishampel DB, Hunt G, Rasskin-Gutman D. Sexual dimorphism in dinosaurs. Dinofest International, Proceedings of a Symposium Sponsored by Arizona State University; 1997. pp. 83–93.

[21] Galton PM. Sex, sacra and *Sellosaurus gracilis* (Saurischia, Sauropodomorpha, Upper Triassic, Germany)—or why the character "two sacral vertebrae" is plesiomorphic for Dinosauria. N Jb Geol Paläont Abh. 1999;213: 19–56.

[22] Barden HE, Maidment SC. Evidence for sexual dimorphism in the stegosaurian dinosaur *Kentrosaurus aethiopicus* from the Upper Jurassic of Tanzania. J Vert Paleontol. 2011;31: 641–651.

[23] Galton PM. *Hypsilophodon foxii* and the other smaller bipedal ornithischian dinosaurs from the Lower Cretaceous of southern England In: Godefroit P, editor. Bernissart Dinosaurs and Early Cretaceous Terrestrial Ecosystems. Bloomington: Indiana University Press; 2012 pp. 225–281.

[24] Colbert EH. The Triassic dinosaur *Coelophysis* . Museum of Northern Arizona Bulletin. 1989;57: 160.

[25] Carpenter K. Variation in *Tyrannosaurus rex* In: Carpenter K, Currie PJ, editors. Dinosaur Systematics: Approaches and Perspectives. Cambridge: Cambridge University Press; 1990 pp. 141–145.

[26] Lü J, Unwin DM, Deeming DC, Jin X, Liu Y, Ji Q. An egg-adult association, gender, and reproduction in pterosaurs. Science. 2011;331: 321–324. 10.1126/science.1197323 21252343

[27] Wang X, Kellner AW, Jiang S, Wang Q, Ma Y, Paidoula Y, et al Sexually dimorphic tridimensionally preserved pterosaurs and their eggs from China. Curr Biol. 2014;24: 1323–1330. 10.1016/j.cub.2014.04.054 24909325

[28] Main RP, Padian K, Horner JR. Comparative histology, growth and evolution of archosaurian osteoderms: why did *Stegosaurus* have such large dorsal plates?. J Vert Paleontol. 2000;20: 56A.

[29] Main R, de Ricqlès A, Horner JR, Padian K. The evolution and function of thyreophoran dinosaur scutes: implications for plate function in stegosaurs. Paleobiology. 2005;31: 291–314.

[30] Padian K, Horner JR. The definition of sexual selection and its implications for dinosaurian biology. J Zool. 2011;283: 23–27.

[31] Padian K, Horner JR. The evolution of ‘bizarre structures’ in dinosaurs: biomechanics, sexual selection, social selection or species recognition?. J Zool. 2011;283: 3–17. 21552308

[32] Padian K, Horner JR. Misconceptions of sexual selection and species recognition: a response to Knell et al. and to Mendelson and Shaw. Trends Ecol Evol. 2013;28: 249–250. 10.1016/j.tree.2013.01.011 23453049

[33] Hone DW, Naish D, Cuthill IC. Does mutual sexual selection explain the evolution of head crests in pterosaurs and dinosaurs?. Lethaia. 2012;45: 139–156.

[34] Chinsamy-Turan A. The Microstructure of Dinosaur Bone: Deciphering Biology with Fine-scale Techniques. Baltimore: Johns Hopkins University Press; 2005.

[35] Hennig E. *Kentrurosaurus aethiopicus*, die stegosaurier-funde vom Tendaguru, Deutsch-Ostafrika. Palaeontographica Supplements. 1925;7: 101–254.

[36] Galton PM. Stegosaurs In: Brett-Surman MK, Holtz TR Jr, Farlow JO, editors. The Complete Dinosaur. 2nd ed. Bloomington: Indiana University Press; 2012 pp. 482–504.

[37] Carpenter K, Miles CA, Cloward K. New primitive stegosaur from the Morrison Formation, Wyoming In: Carpenter K, editor. The Armored Dinosaurs. Bloomington: Indiana University Press; 2001 pp. 55–75.

[38] Maidment SC, Norman DB, Barrett PM, Upchurch P. Systematics and phylogeny of Stegosauria (Dinosauria: Ornithischia). J Syst Palaeontol. 2008;6: 367–407.

[39] Carpenter K. Species concept in North American stegosaurs. Swiss J Geosci. 2010;103: 155–162.

[40] Ayer JV. The Howe Ranch Dinosaurs. Aathal, Switzerland: Sauriermuseum Aathal; 2000 pp. 95.

[41] Schmude DE, Weege CJ. Stratigraphic relationship, sedimentology, and taphonomy of Meilyn, a dinosaur quarry in the basal Morrison Formation of Wyoming. In: Morales M, editor. The Continental Jurassic. Museum of Northern Arizona Bulletin. 1996;60: 547–554.

[42] Hayashi S, Carpenter K, Suzuki D. Different growth patterns between the skeleton and osteoderms of *Stegosaurus* (Ornithischia: Thyreophora). J Vert Paleontol. 2009;29: 123–131.

[43] Redelstorff R, Sander PM. Long and girdle bone histology of *Stegosaurus*: implications for growth and life history. J Vert Paleontol. 2009;29: 1087–1099.

[44] Hayashi S, Carpenter K, Watabe M, McWhinney LA. Ontogenetic histology of *Stegosaurus* plates and spikes. Palaeontology. 2012;55: 145–161.

[45] Voorhies MR. Taphonomy and population dynamics of an early Pliocene vertebrate fauna, Knox County, Nebraska. Rocky Mountain Geology. 1969;8: 1–69.

[46] Hunt R, Farke A. Behavioral interpretations from ceratopsid bonebeds In: Ryan MJ, Chinnery-Allgeier BJ, Eberth DA, editors. New Perspectives on Horned Dinosaurs: The Royal Tyrrell Museum Ceratopsian Symposium. Bloomington: Indiana University Press; 2010 pp. 447–455

[47] Richmond DR, Morris TH. The dinosaur death-trap of the Cleveland Lloyd Quarry, Emery County, Utah. Museum of Northern Arizona Bulletin. 1996;60: 533–545.

[48] Packer C. Sexual dimorphism: the horns of African antelopes. Science. 1983;221: 1191–1193. 17811523 10.1126/science.221.4616.1191

[49] Bro‐Jørgensen J. The intensity of sexual selection predicts weapon size in male bovids. Evolution. 2007;61: 1316–1326. 17542842 10.1111/j.1558-5646.2007.00111.x

[50] Stankowich T, Caro T. Evolution of weaponry in female bovids. Proc R Soc Lond B Biol Sci. 2009;276: 4329–4334. 10.1098/rspb.2009.1256 PMC2817105 19759035

[51] Carpenter K, Sanders F, McWhinney LA, Wood L. Evidence for predator-prey relationships In: Carpenter K, editor. The Carnivorous Dinosaurs. Bloomington: Indiana University Press; 2005 pp. 325–350.

[52] Mallison H. Defence capabilities of *Kentrosaurus aethiopicus* Hennig, 1915. Palaeontol Electron. 2011;14: 1–25.

[53] Horner JR, Goodwin MB. Major cranial changes during *Triceratops* ontogeny. Proc R Soc Lond B Biol Sci. 2006;273: 2757–2761. 10.1098/rspb.2006.3643 PMC1635501 17015322

[54] Maidment SC, Linton DH, Upchurch P, Barrett PM. Limb-bone scaling indicates diverse stance and gait in quadrupedal ornithischian dinosaurs. PLOS one. 2012;7: e36904 10.1371/journal.pone.0036904 22666333 PMC3358279

[55] Gilmore CW. Osteology of the armoured Dinosauria in the United States National Museum, with special reference to the genus *Stegosaurus* . United States National Museum Bulletin. 1914;89: 1–143.

[56] Paul GS. The science and art of restoring the life appearance of dinosaurs and their relatives In: Czerkas SJ, Olson EC, editors. Dinosaurs Past and Present, Volume 2 Seattle: University of Washington Press; 1987 pp. 4–49.

[57] Paul GS. The arrangement of plates in the first complete *Stegosaurus*, from Garden Park. Garden Park Paleontological Society, Tracks in Time. 1992;3: 1–2.

[58] Carpenter K. How to make a fossil: part 1—fossilizing bone. Journal of Paleontological Sciences. 2007;JPS.C.07.0001. pp. 1–10.

