# Supplementary figures and images for: Evidence for Sexual Dimorphism in the Plated Dinosaur Stegosaurus mjosi (Ornithischia, Stegosauria) from the Morrison Formation (Upper Jurassic) of Western USA
Source: PLoS One. 2015 Apr 22;10(4):e0123503. doi: 10.1371/journal.pone.0123503 (PMC4406738; doi:10.1371/journal.pone.0123503)

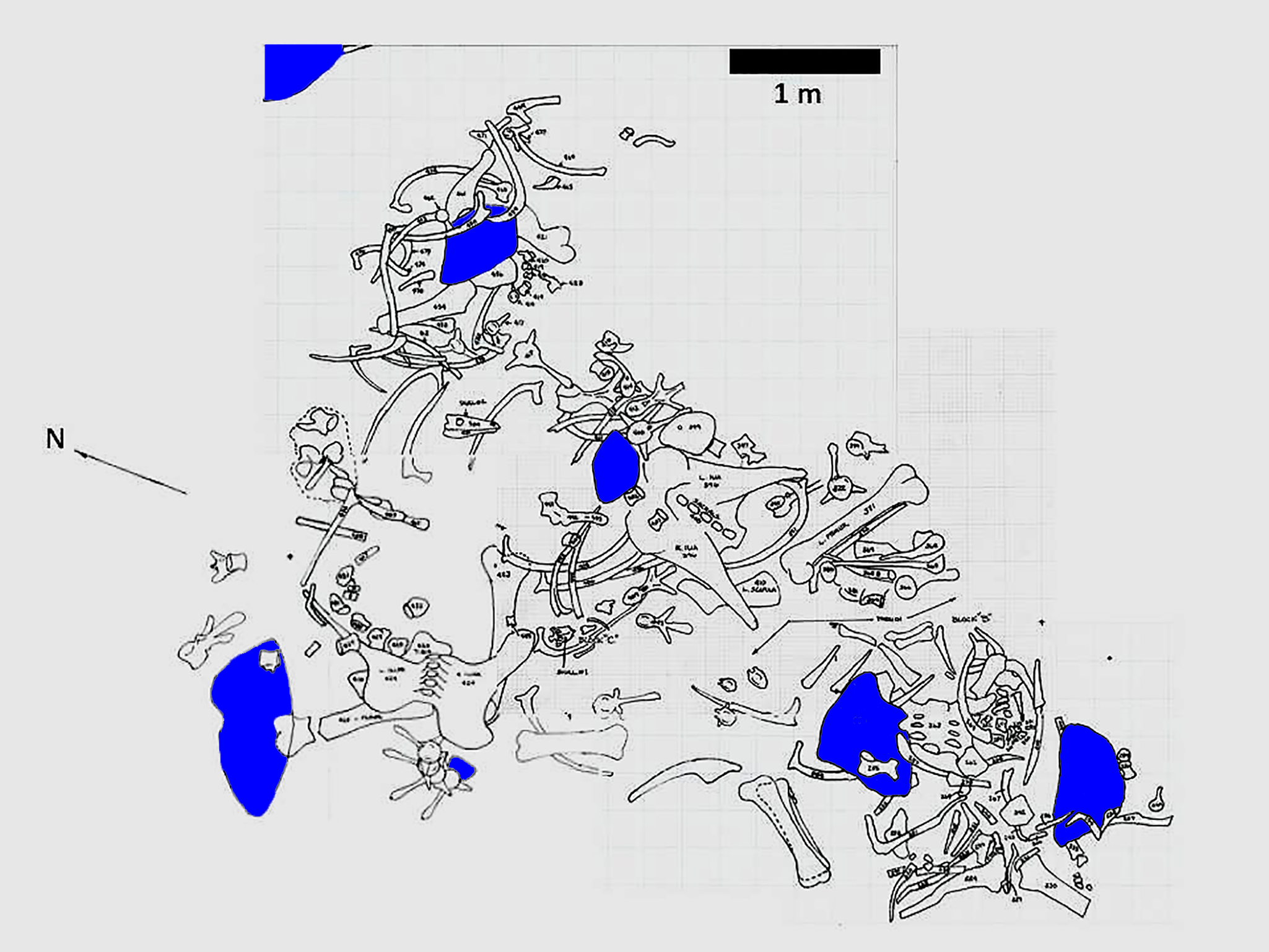

Supplement: S1 Fig — Direction of modern north is indicated. Fairly complete plates are indicated in blue. As fieldwork has continued in the quarry, a full map has yet to be completed. Therefore, some bones in the northern portion of the quarry have not yet been included. Courtesy of the Judith River Dinosaur Institute. (TIF) [file pone.0123503.s007.tif]

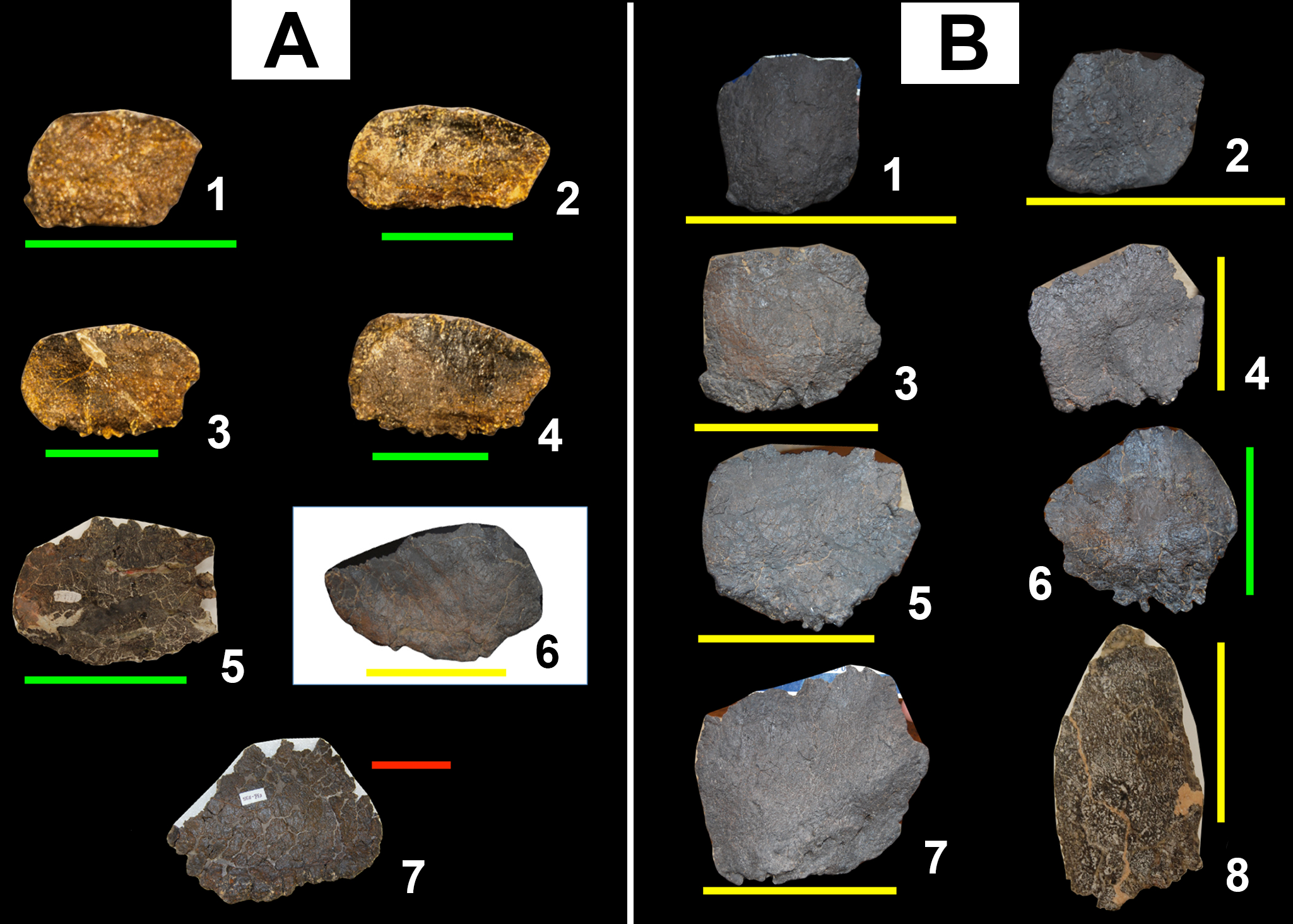

Supplement: S2 Fig — (A) Wide morph cervical plates. (1–4) HMNS 14. (5) WDC DMQ-001; J9979. (6) VFSMA 001. (7) JRDI 5ES-553. Images of HMNS 14 courtesy of K. Carpenter. (B) Tall morph cervical plates. (1–7) SMA 0092. (8) WDC DMQ-001; 9791. Plates are hypothetically ordered from anterior-most to posterior-most, although the numbers are not meant to indicate precise plate position and certain plate positions are probably duplicated in the sample. Some images are flipped so the anterior edge is to the left. Color of scale bar (= 10cm) indicates level of completeness. Green—Plates that are complete enough for an accurate outline to be reconstructed. Yellow—Plates that are not entirely complete, but allow for a plausible outline to be reconstructed. Red—Plates that are incomplete and from which an outline cannot be reconstructed. (TIF) [file pone.0123503.s008.tif]

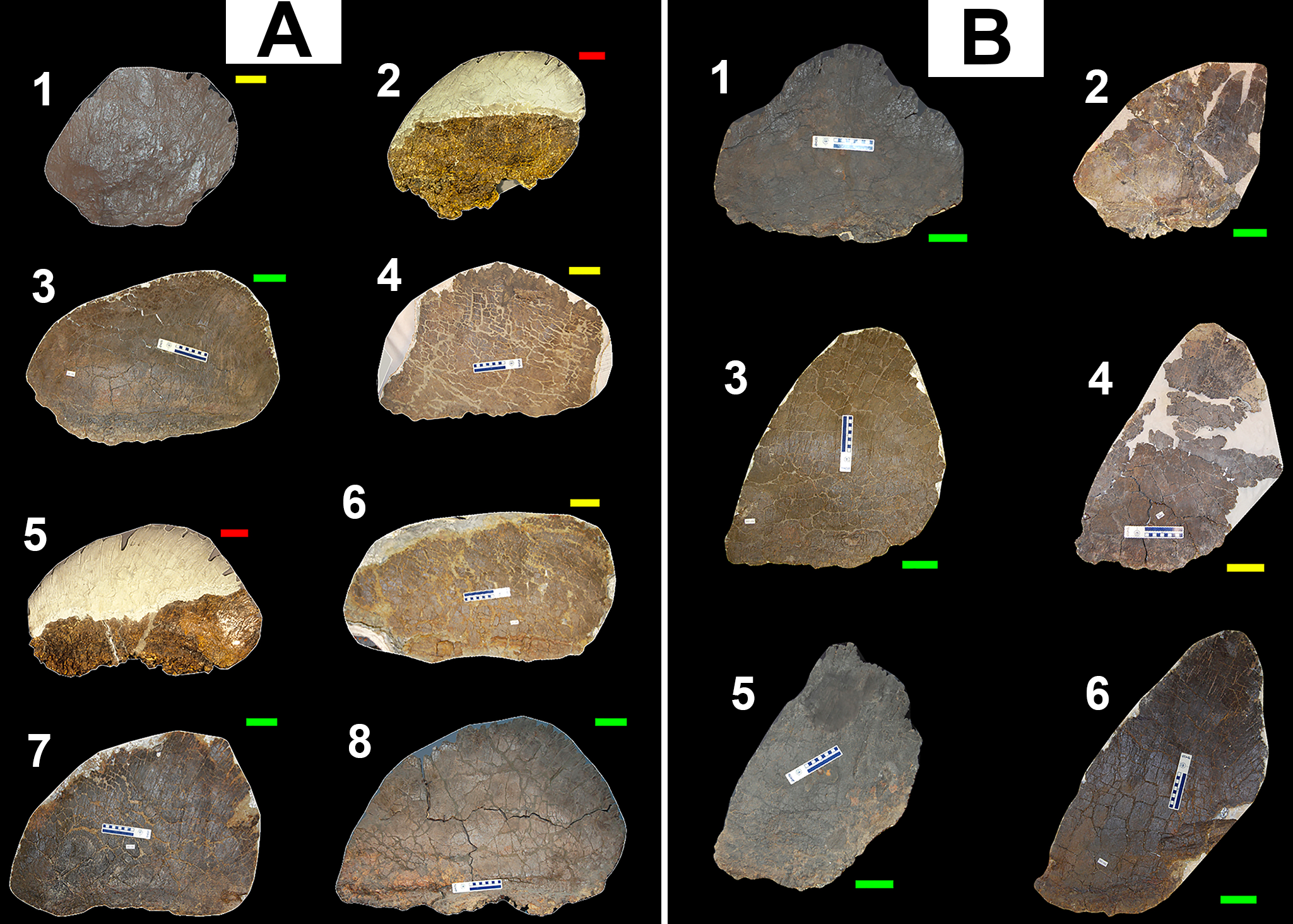

Supplement: S3 Fig — (A) Wide morph dorsal plates. (1) Cast of VFSMA 001. (2) HMNS 14. (3) JRDI 5ES-523. (4) VFSMA 001. (5) HMNS 14. (6) JRDI 5ES-518. (7) JRDI 5ES-256. (8) SMA 0018. Images of HMNS 14 courtesy of K. Carpenter. (B) Tall morph dorsal plates. (1) SMA 0092 that was found to be an outlier in PCA. (2) WDC DMQ-001; from block 9999. (3) JRDI 5ES-237. (4) JRDI 5ES-357. (5) SMA 0092. (6) JRDI 5ES-552. Plates are hypothetically ordered from anterior-most to posterior-most, although the numbers are not meant to indicate precise plate position and certain plate positions are probably duplicated in the sample. Some images are flipped so the anterior edge is to the left. Color of scale bars (= 10 cm) as in S2 Fig. (TIF) [file pone.0123503.s009.tif]

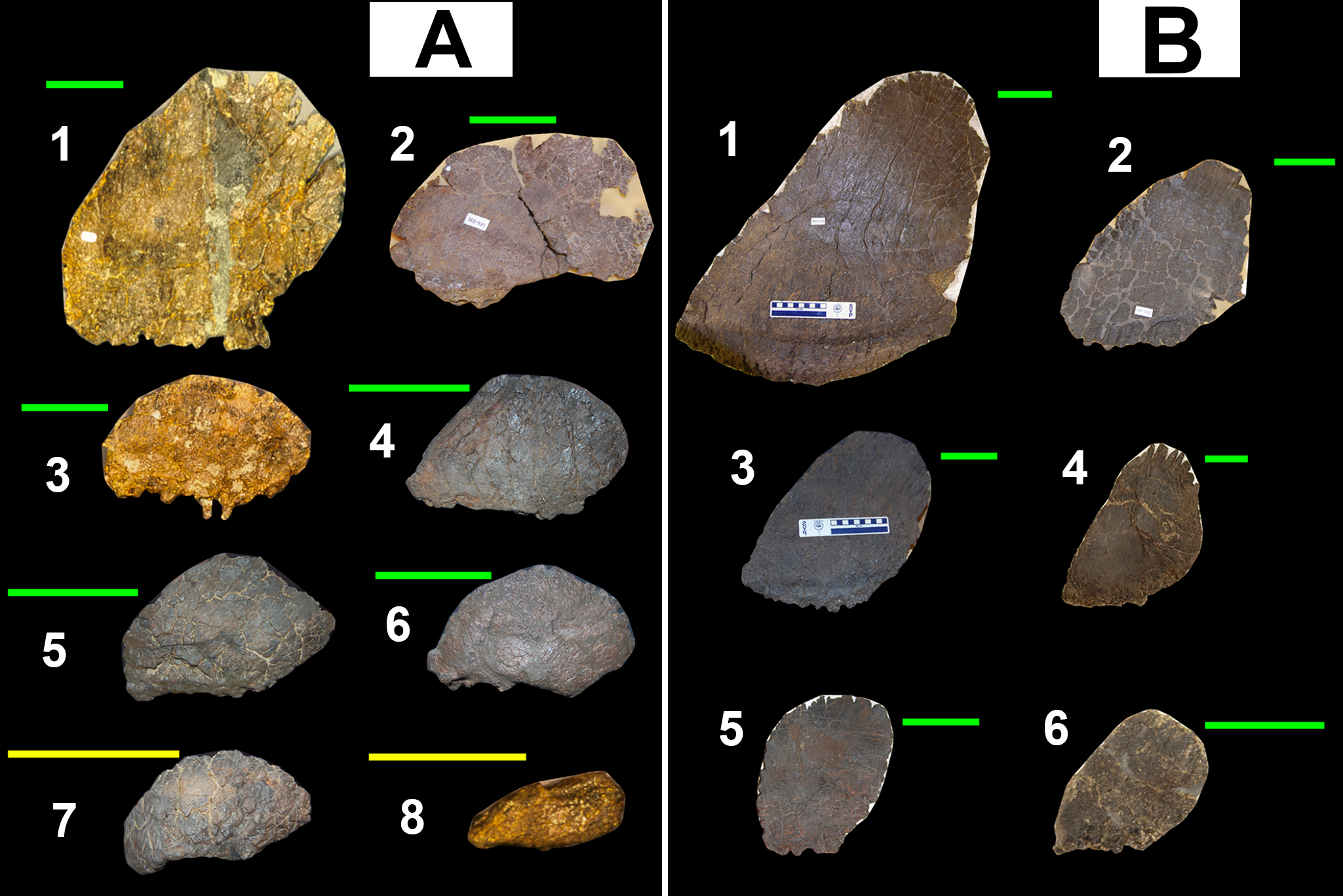

Supplement: S4 Fig — (A) Wide morph caudal plates. (1) HMNS 14. (2) JRDI 5ES-525. (3) HMNS 14. (4, 5) VFSMA 001. (6) SMA 0018. (7) VFSMA 001. (8) HMNS 14. Images of HMNS 14 courtesy of K. Carpenter. (B) Tall morph caudal plates. (1) JRDI 5ES-579. (2) JRDI 5ES-401. (3) SMA 0092. (4) WDC DMQ-001; 9808. (5) SMA 0092. (6) WDC DMQ-001; 570P. Plates are hypothetically ordered from anterior-most to posterior-most, although the numbers are not meant to indicate precise plate position and certain plate positions are probably duplicated in the sample. Some images are flipped so the anterior edge is to the left. Color of scale bars (= 10 cm) as in S2 Fig. (TIF) [file pone.0123503.s010.tif]

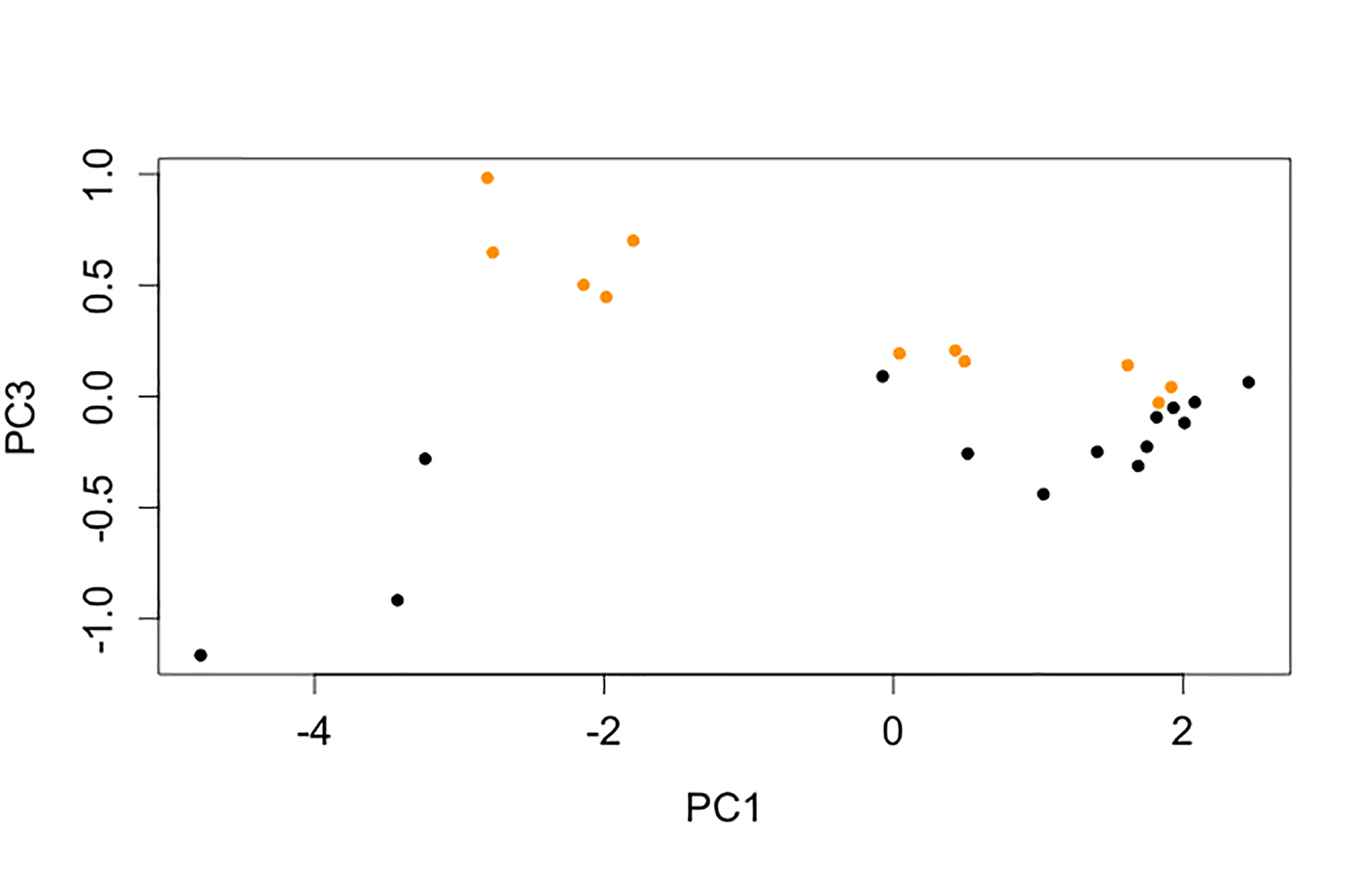

Supplement: S5 Fig — Orange and black points are plates identified to be of the tall and wide morph, respectively. Decreasing values of PC1 indicate larger perimeter, surface area, and base length. With decreasing PC1 values, tall morph plate variation follows a trend of narrowing ‘width’ and increasing distance between base center and apex, while wide morph plate variation follows a trend of increasing ‘width’ (n = 25). (TIF) [file pone.0123503.s011.tif]

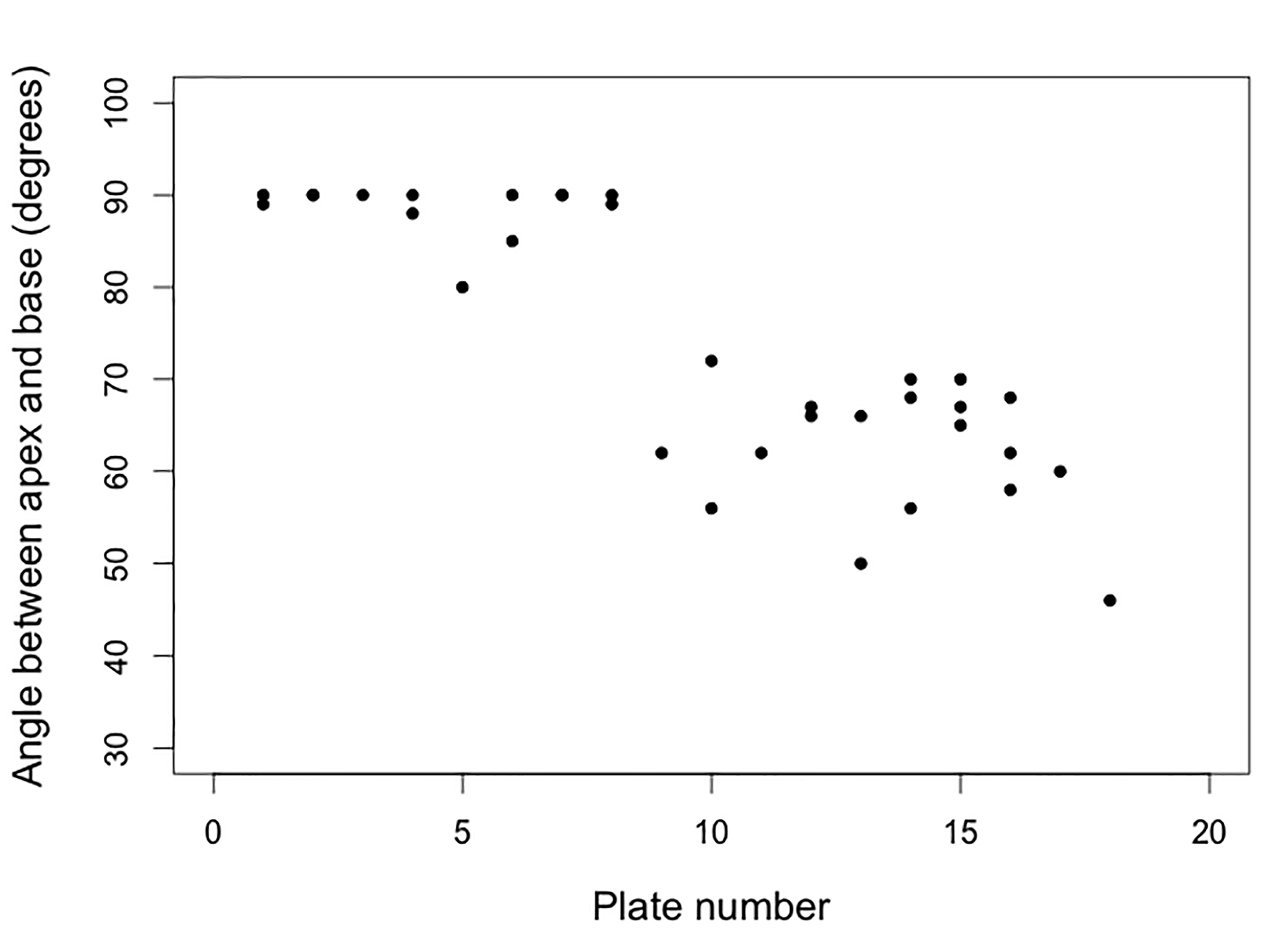

Supplement: S6 Fig — The data for articulated specimens of Stegosaurus: NHMUK R36730, DMNS 2818, and USNM 4934. Plate numbers start at the anterior of the specimen and increase posteriorly. Highly incomplete plates or plates that are entirely missing from the specimen are not included in the plot (n = 33). (TIF) [file pone.0123503.s012.tif]

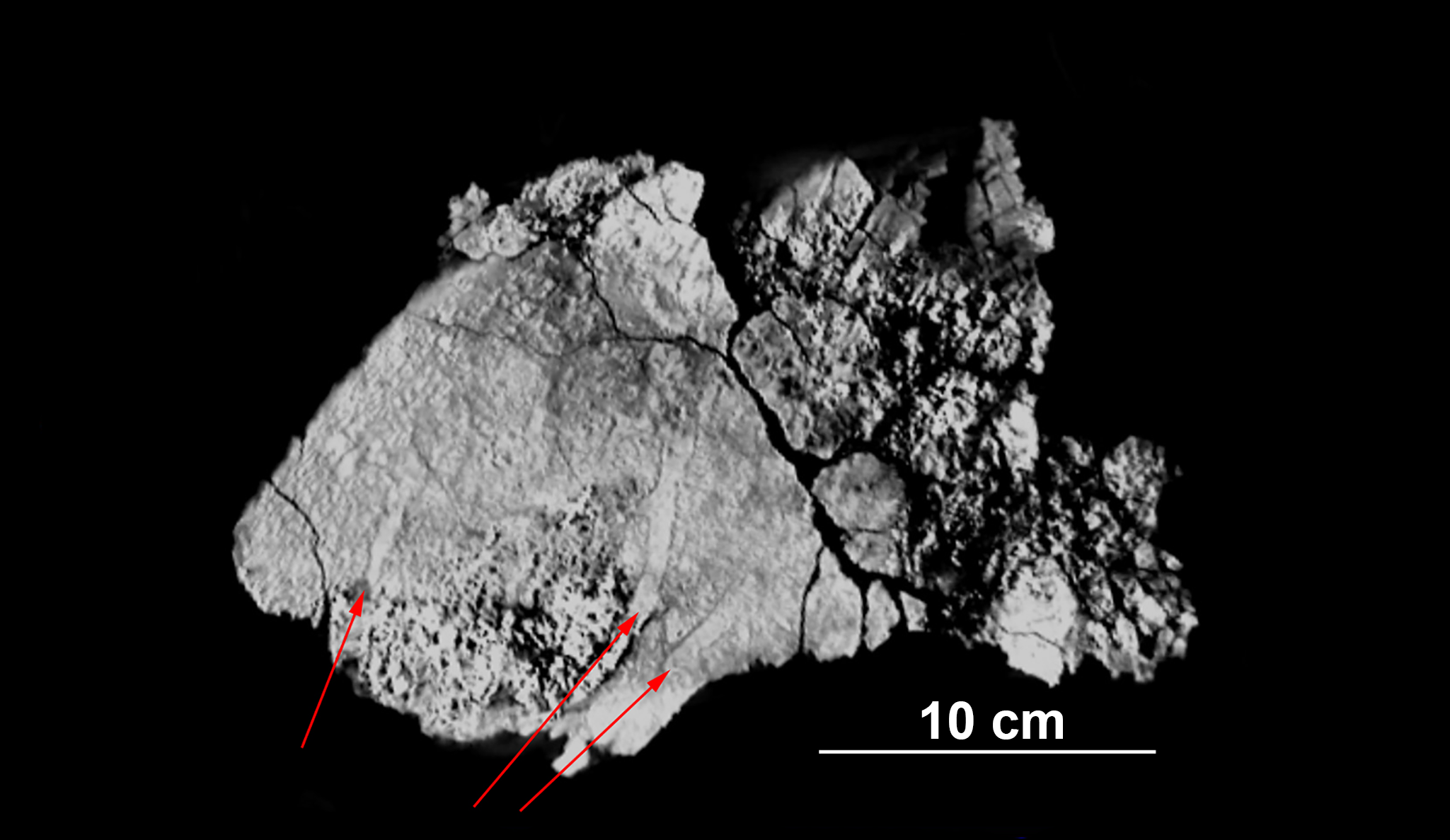

Supplement: S7 Fig — Red arrows indicate internal vascular piping. (TIF) [file pone.0123503.s013.tif]

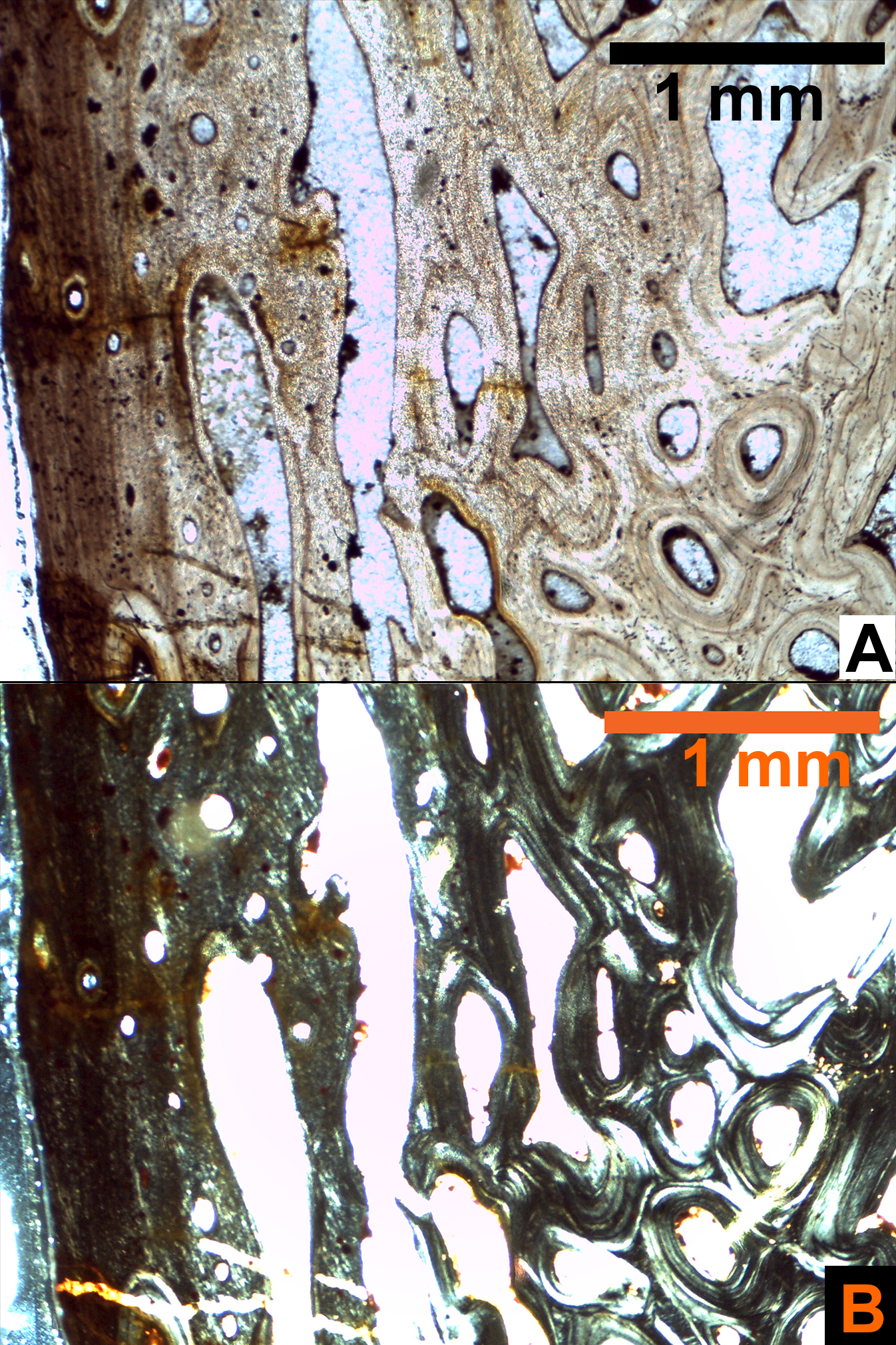

Supplement: S8 Fig — (A) Under plane polarized light. (B) Under crossed polarized light. Bone surface is to the left. Note presence of EFS. (TIF) [file pone.0123503.s014.tif]

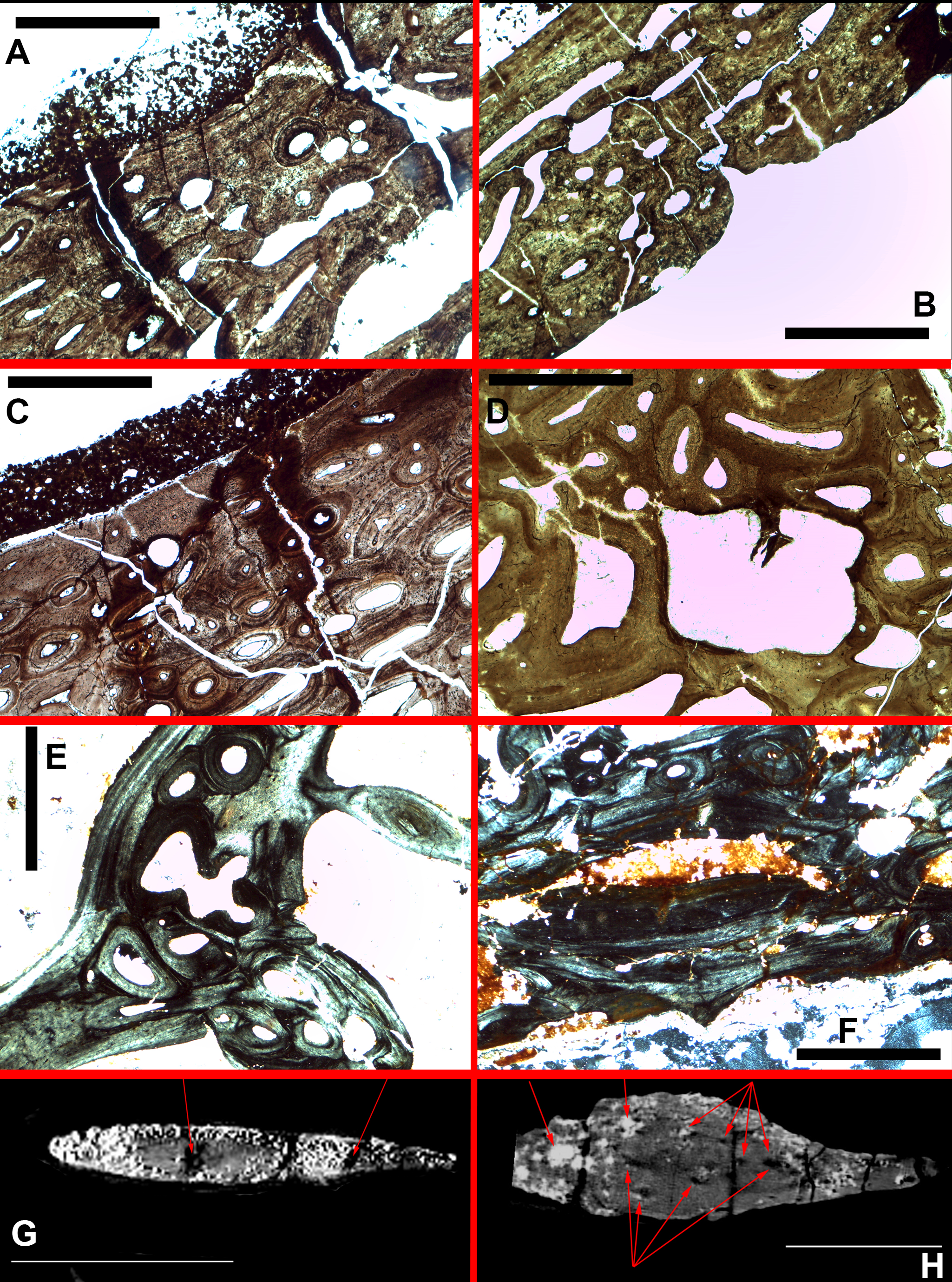

Supplement: S9 Fig — (A, B) Apex cortical bone under plane polarized light. Midplate cortical (C) and cancellous (D) bone under plane polarized light. Base cancellous (E) and cortical (F) bone under crossed polarized light (Scale bars = 1 mm). Bone surface is towards scale bar in A-C, F. CT cross sections along the transverse (G) and frontal (H) planes. Red arrows indicate internal vascular piping (Scale bars = 10 cm). (TIF) [file pone.0123503.s015.tif]

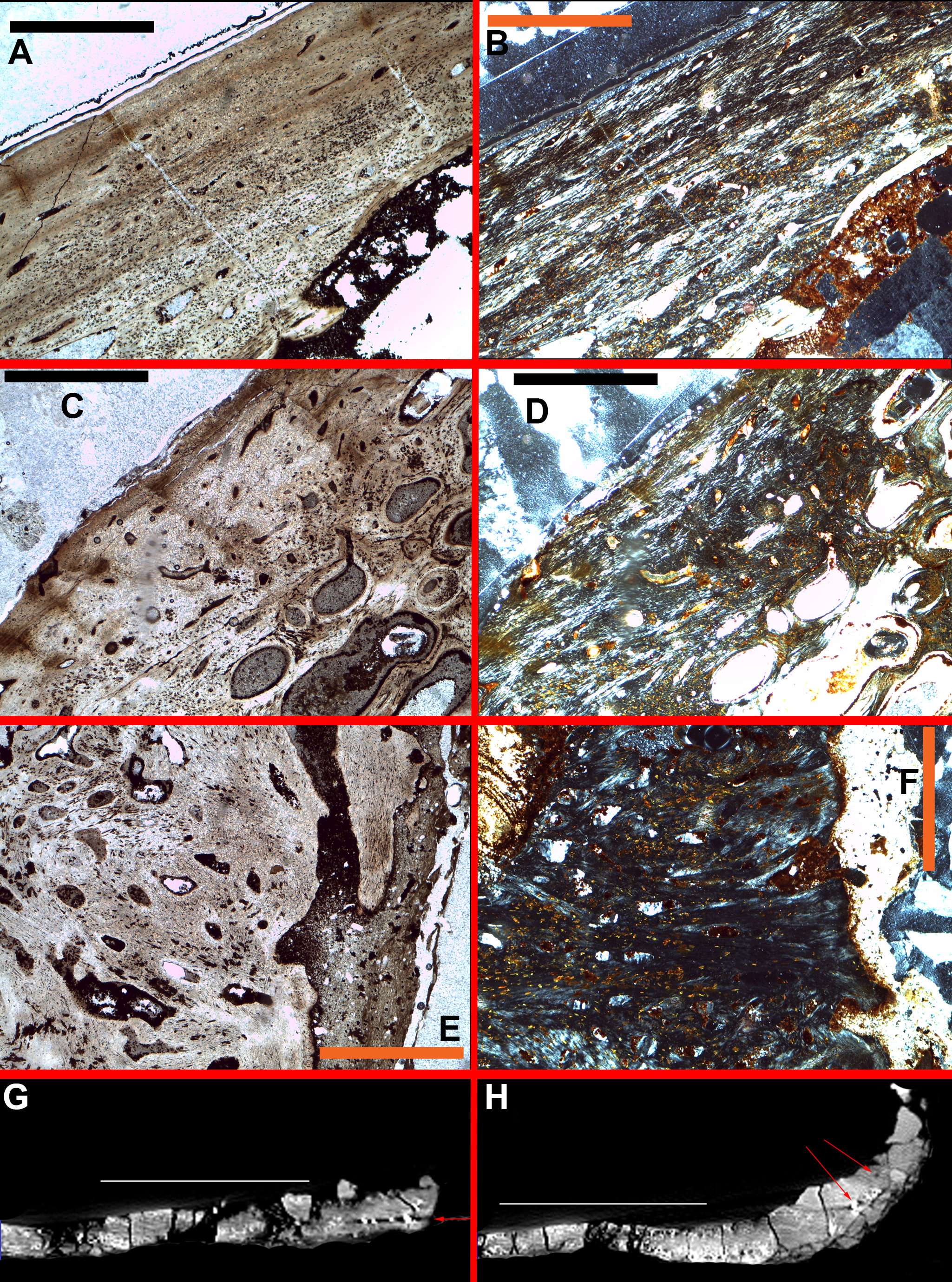

Supplement: S10 Fig — The same image of apex cortical bone under (A) plane polarized and (B) crossed polarized light. The same image of midplate cortical bone under (C) plane polarized and (D) crossed polarized light. Base cortical bone under (E) plane polarized and (F) crossed polarized light (Scale bars = 1 mm). Bone surface is towards scale bar in A-F. (G, H) CT cross sections along the transverse plane. Red arrows indicate internal vascular piping (Scale bars = 10 cm). (TIF) [file pone.0123503.s016.tif]

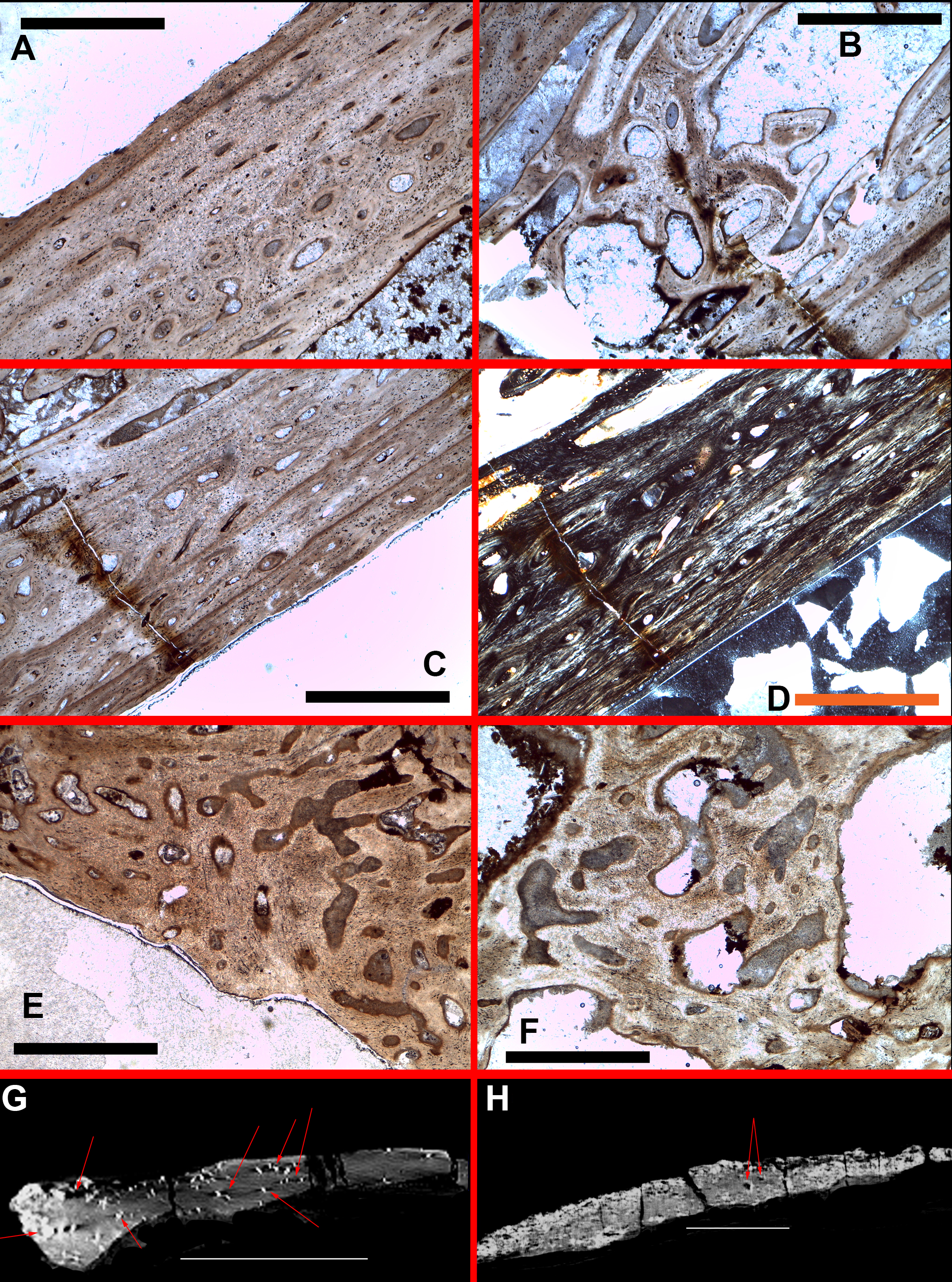

Supplement: S11 Fig — Apex cortical (A) and cancellous (B) bone under plane polarized light. The same image of midplate cortical bone under (C) plane polarized and (D) crossed polarized light. Base cortical (E) and cancellous (F) bone under plane polarized light (Scale bars = 1 mm). Bone surface is towards scale bar in A, C-E. CT cross sections along the transverse (G) and frontal (H) plane. Red arrows indicate internal vascular piping (Scale bars = 10 cm). (TIF) [file pone.0123503.s017.tif]

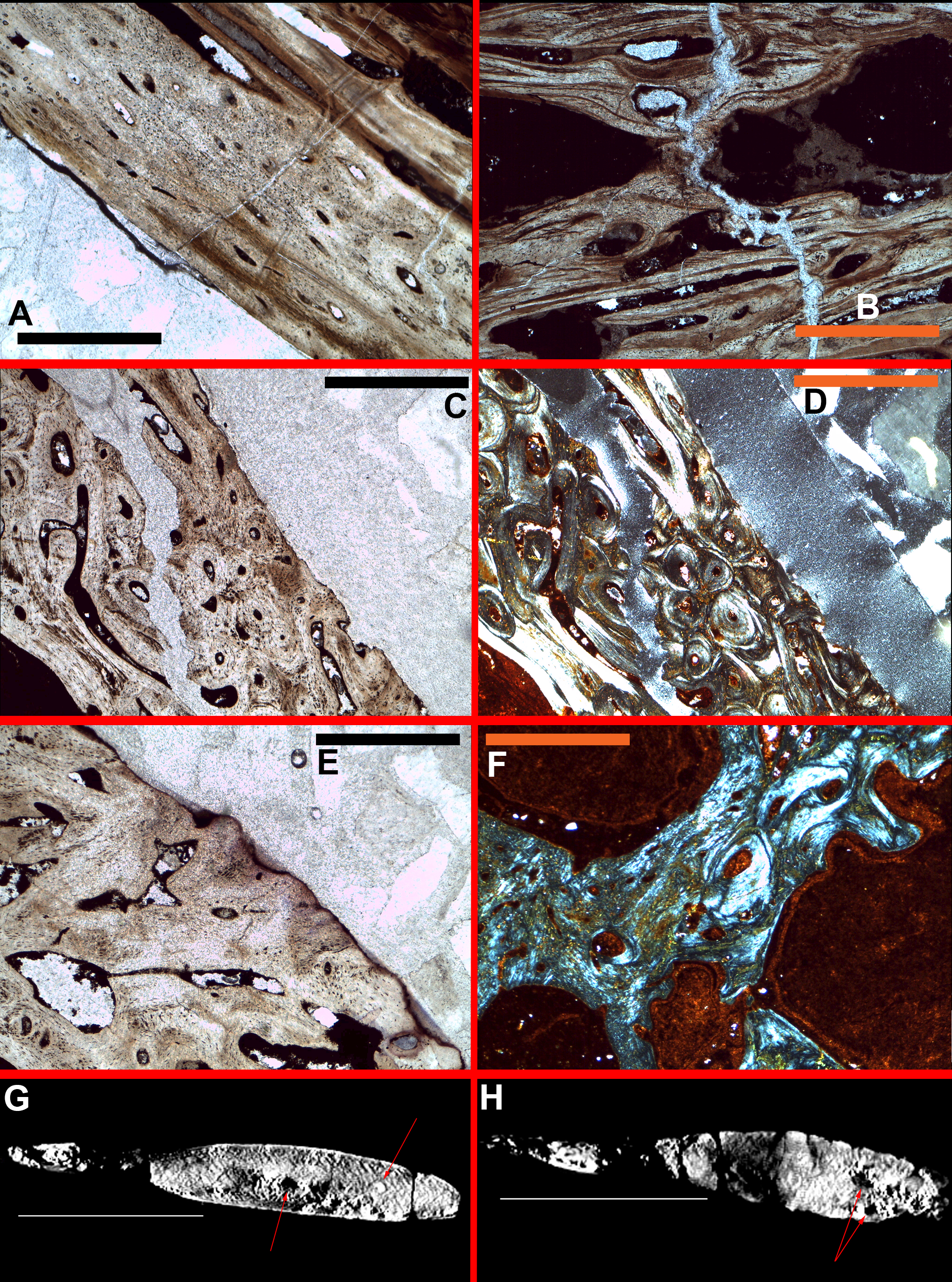

Supplement: S12 Fig — Apex cortical (A) and cancellous (B) bone under plane polarized light. The same image of midplate cortical bone under (C) plane polarized and (D) crossed polarized light. Base cortical (E) and cancellous (F) bone under plane polarized and crossed polarized light, respectively (Scale bars = 1 mm). Bone surface is towards scale bar in A, C-E. (G, H) CT cross sections along the frontal plane. Red arrows indicate internal vascular piping (Scale bars = 10 cm). (TIF) [file pone.0123503.s018.tif]

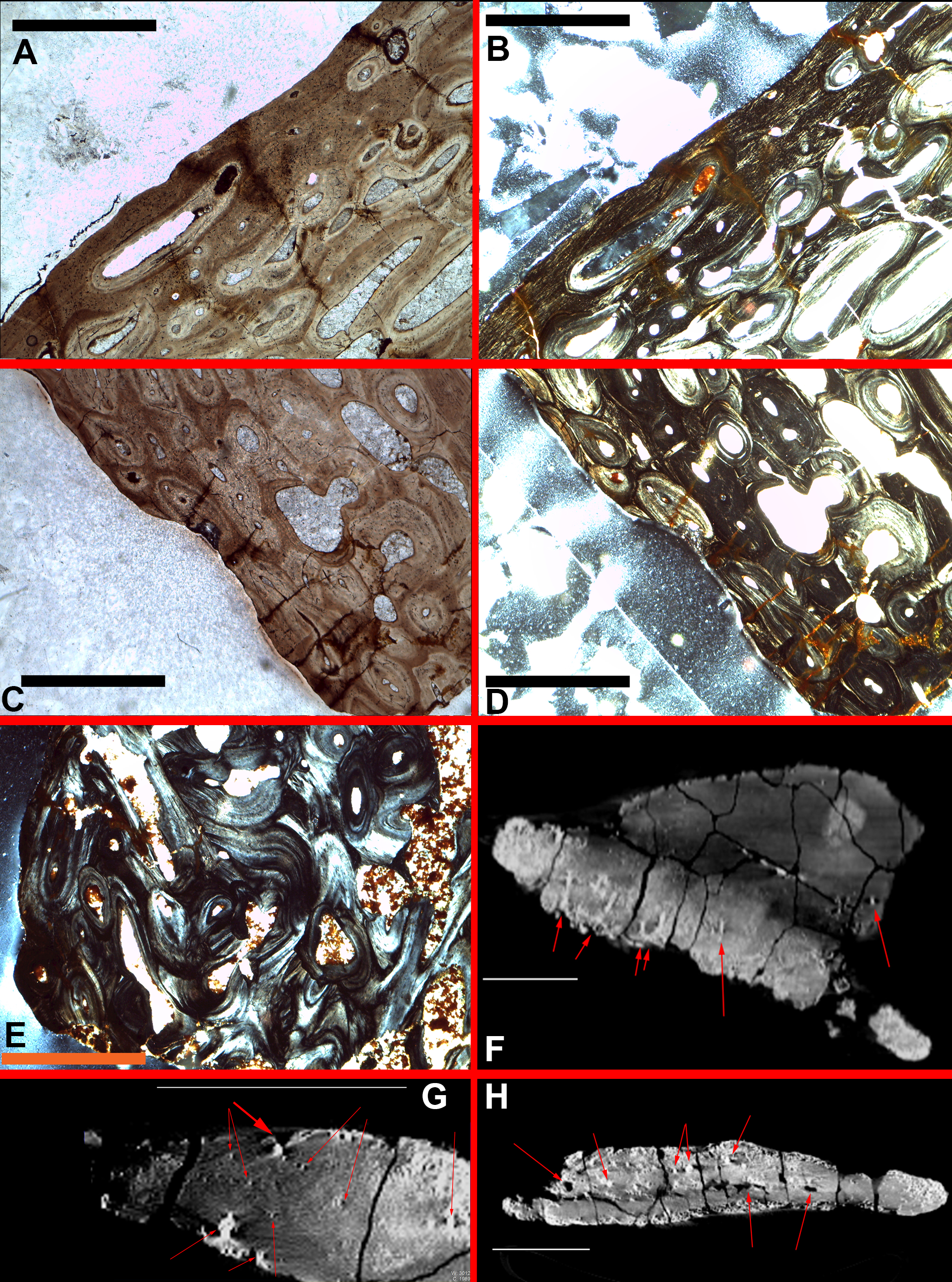

Supplement: S13 Fig — The same image of apex cortical bone under (A) plane polarized and (B) crossed polarized light. The same image of midplate cortical bone under (C) plane polarized and (D) crossed polarized light. (E) Base cortical bone under crossed polarized light (Scale bars = 1 mm). Bone surface is towards scale bar in A-E. CT cross sections along the (F) sagittal, (G) frontal, and (H) transverse plane. Red arrows indicate internal vascular piping. Large red arrow in G indicates pipe exiting onto surface of the plate (Scale bars = 10 cm). (TIF) [file pone.0123503.s019.tif]

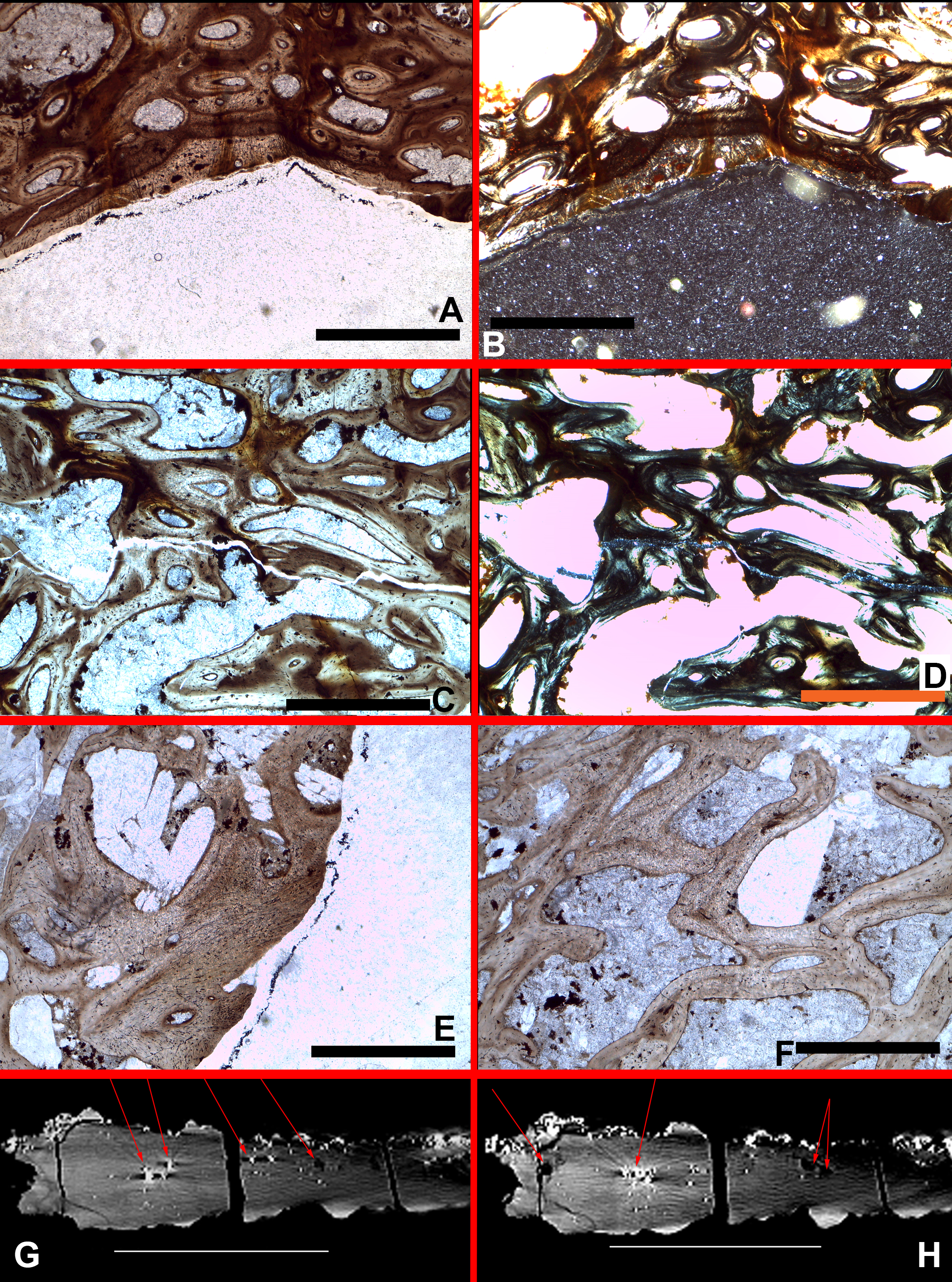

Supplement: S14 Fig — The same image of midplate cortical bone under (A) plane polarized and (B) crossed polarized light. The same image of midplate cancellous bone under (C) plane polarized and (D) crossed polarized light. Base cortical (E) and cancellous (F) bone under plane polarized light (Scale bars = 1 mm). Bone surface is towards scale bar in A, B, E. (G, H) CT cross sections along the transverse plane. Red arrows indicate internal vascular piping (Scale bars = 10 cm). (TIF) [file pone.0123503.s020.tif]

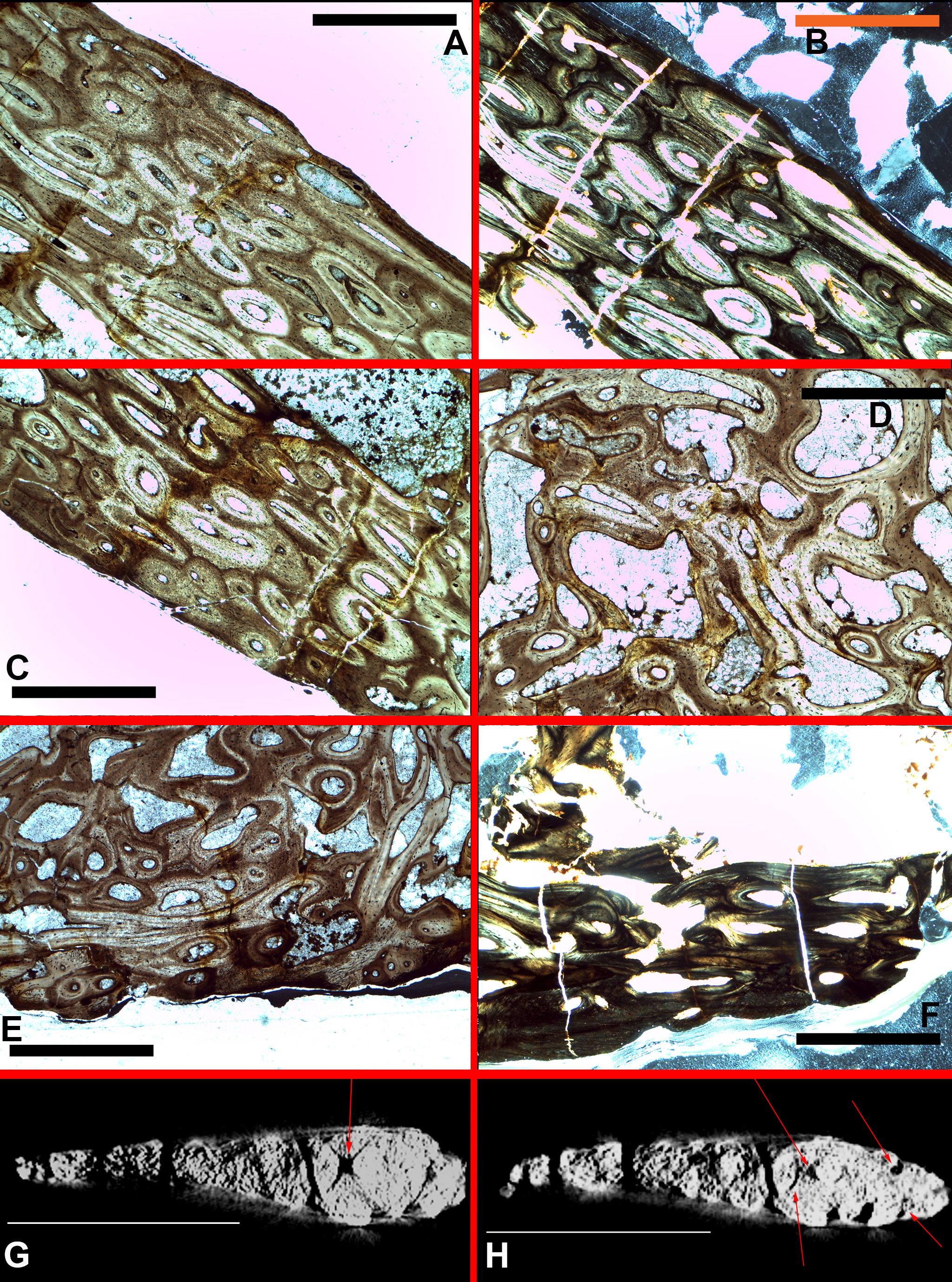

Supplement: S15 Fig — The same image of apex cortical bone under (A) plane polarized and (B) crossed polarized light. (C) Apex cortical bone under plane polarized light. Midplate cancellous (D) and cortical (E) bone under plane polarized light. Base cortical (F) bone under crossed polarized light (Scale bars = 1 mm). Bone surface is towards scale bar in A-C, E, F. CT cross sections along the transverse (G) and frontal (H) plane. Red arrows indicate internal vascular piping (Scale bars = 10 cm). (TIF) [file pone.0123503.s021.tif]

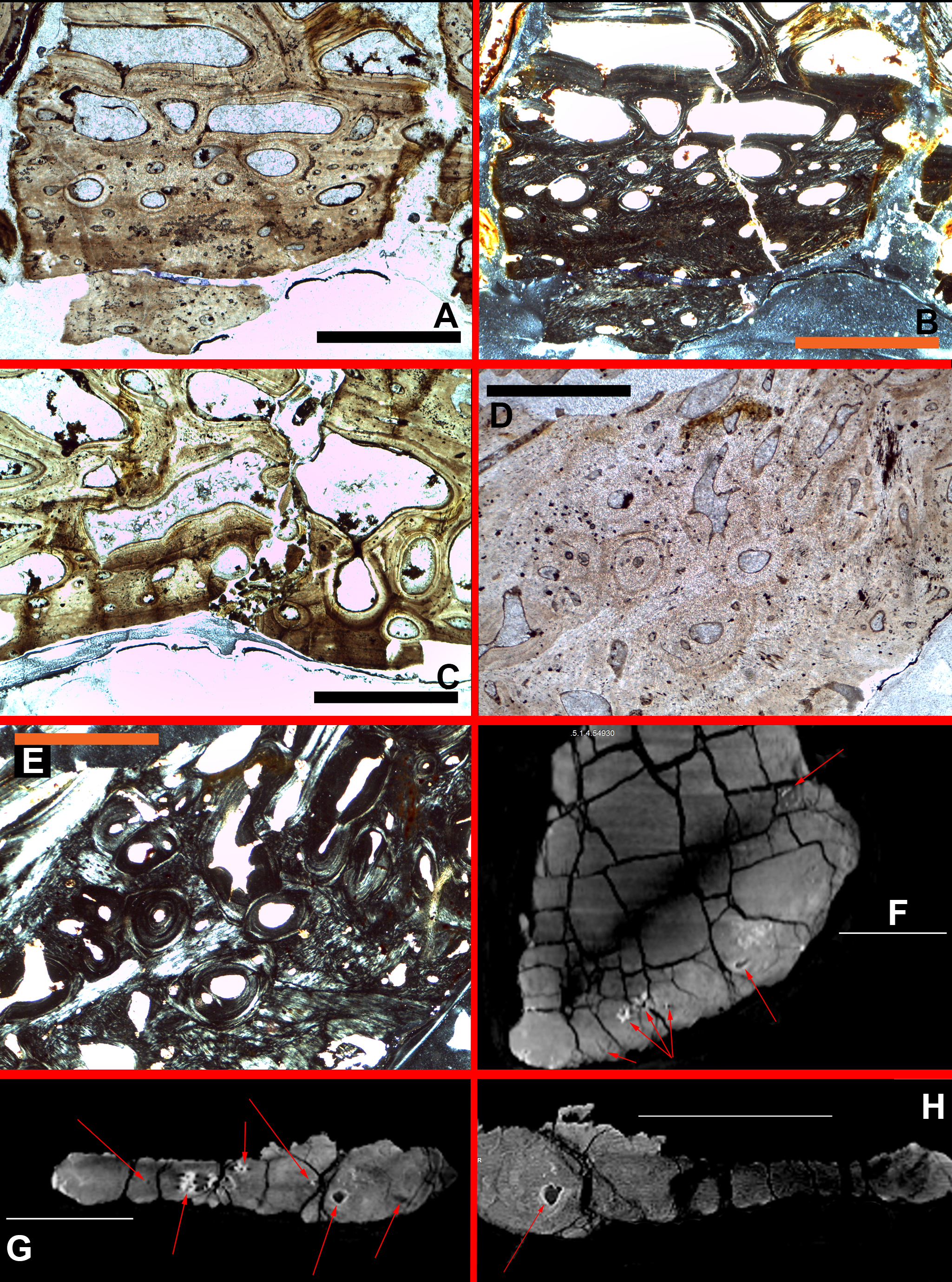

Supplement: S16 Fig — The same image of apex cortical bone under (A) plane polarized and (B) crossed polarized light. (C) Apex cortical bone under plane polarized light. The same image of base cortical bone under (D) plane polarized and (E) crossed polarized light (Scale bars = 1 mm). Bone surface is towards scale bar in A-C, but are opposite to the scale bar in D, E. CT cross sections along the sagittal (F), transverse (G), and frontal (H) plane. Red arrows indicate internal vascular piping (Scale bars = 10 cm). (TIF) [file pone.0123503.s022.tif]

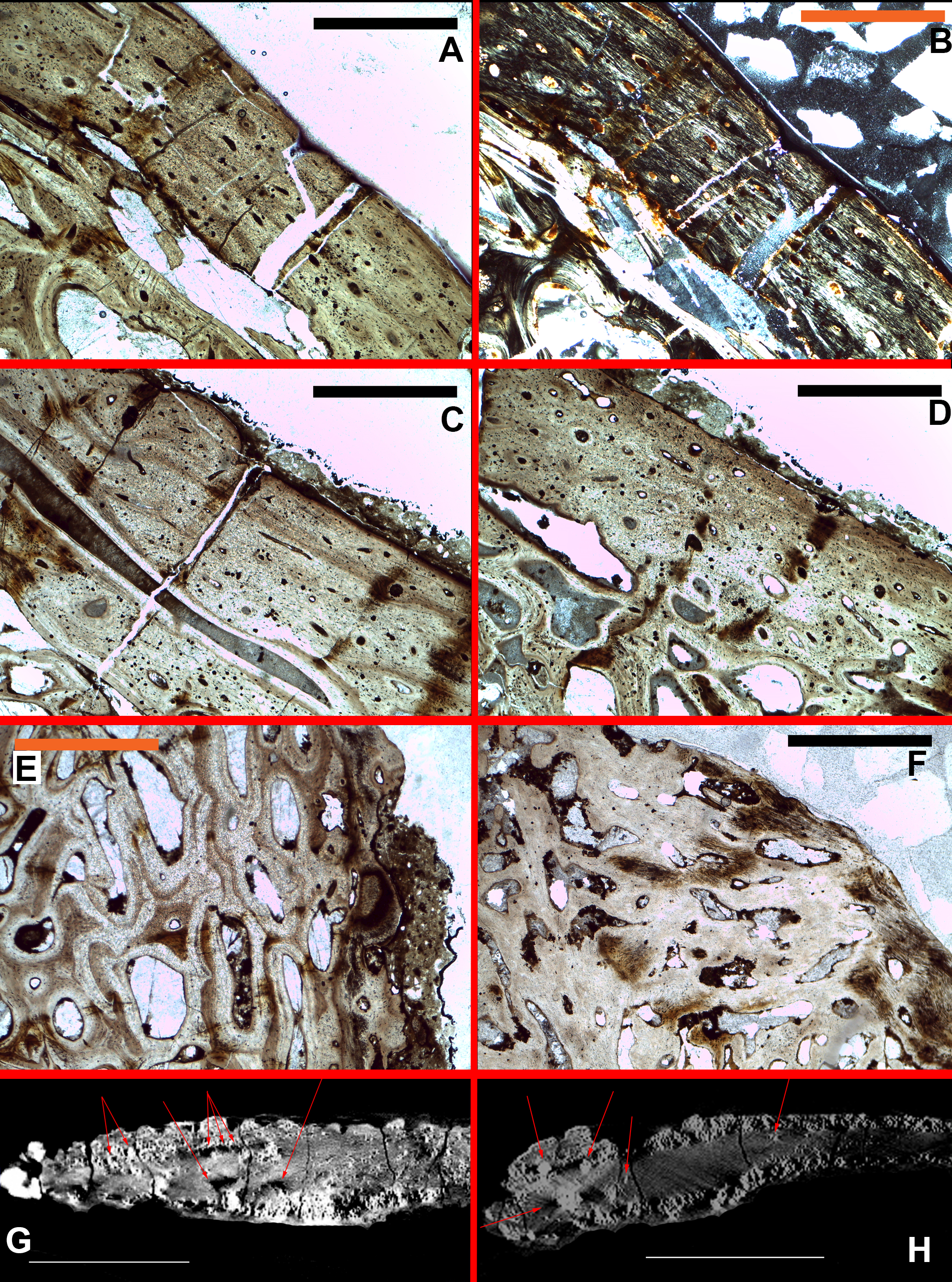

Supplement: S17 Fig — The same image of apex cortical bone under (A) plane polarized and (B) crossed polarized light. Apex (C, D), midplate (E), and base (F) cortical bone under plane polarized light (Scale bars = 1 mm). Bone surface is towards scale bar in A-D, F but is opposite to the scale bar in E. CT cross sections along the frontal (G) and transverse (H) plane. Red arrows indicate internal vascular piping (Scale bars = 10 cm). (TIF) [file pone.0123503.s023.tif]

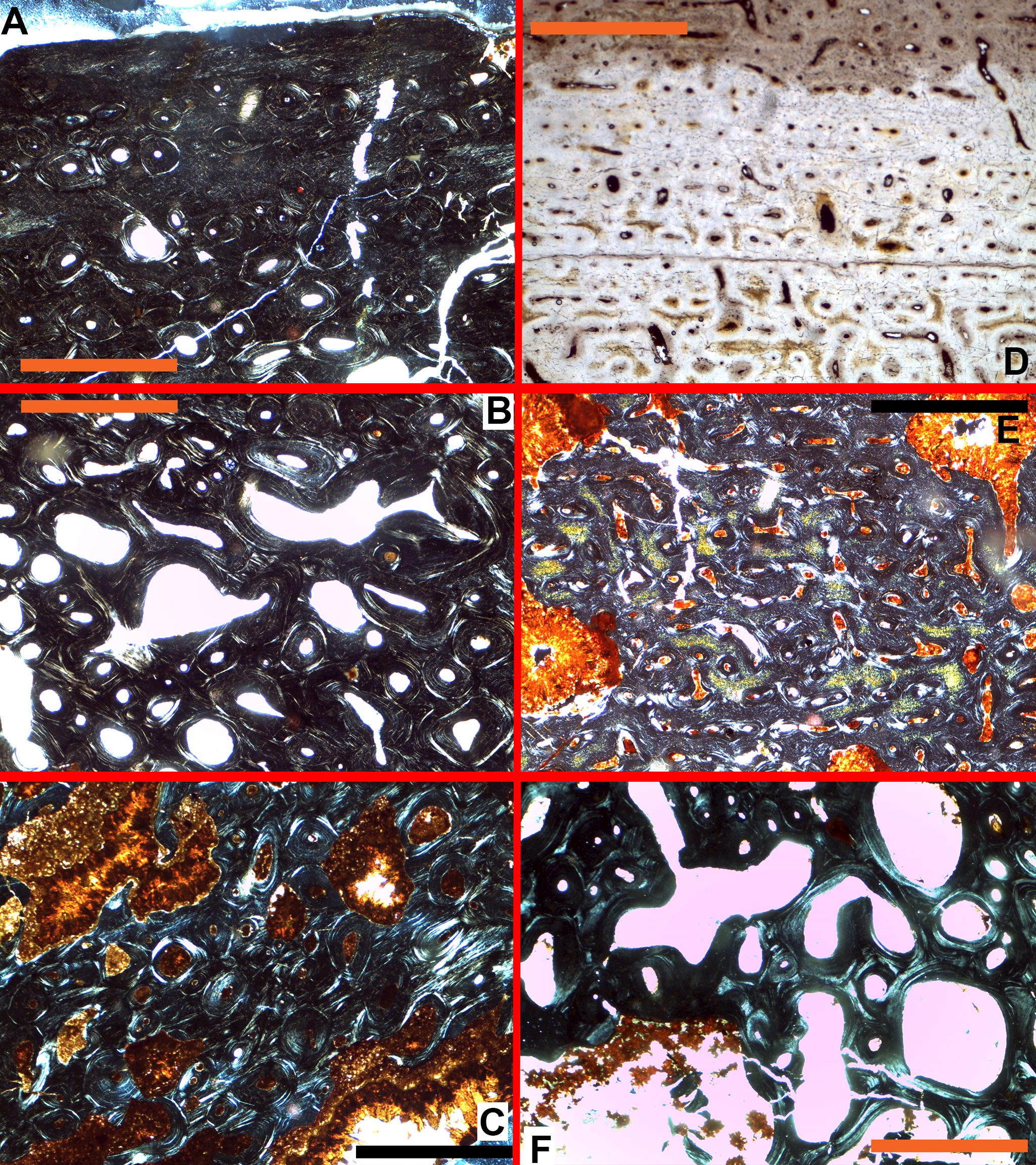

Supplement: S18 Fig — The femur JRDI 5ES-229 histology from the outer (A), middle (B), and inner (C) regions under crossed polarized light. The tibia JRDI 5ES-501 histology from the outer (D), middle (E), and inner (F) regions. D is under plane polarized light while D, F are under crossed polarized light (Scale bars = 1 mm). Periosteal surface is towards the top and endosteal surface is towards the bottom in all images. (TIF) [file pone.0123503.s024.tif]

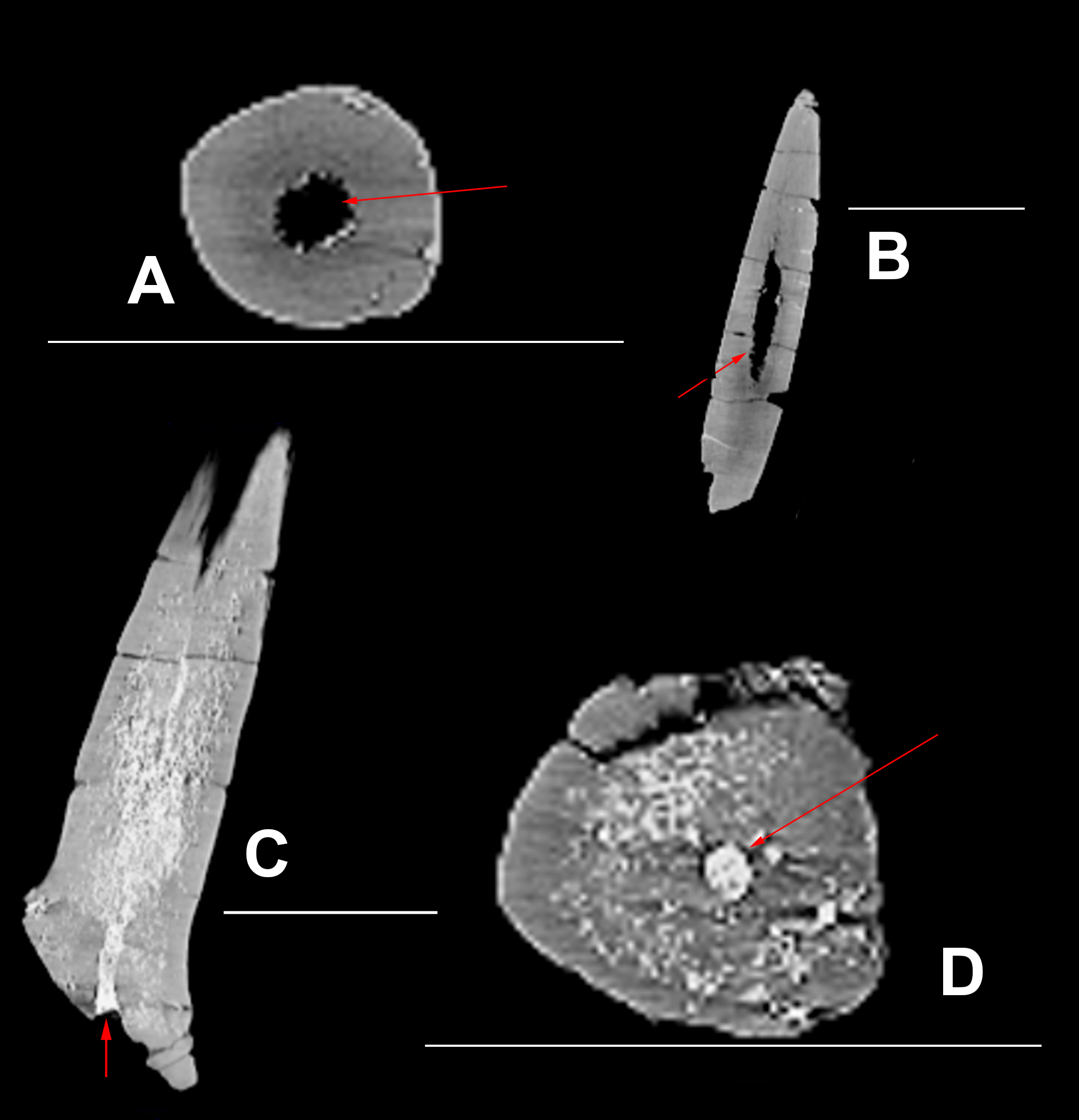

Supplement: S19 Fig — Cross sections of posterior spikes (A) JRDI 5ES-245 and (B) JRDI 5ES-258 along the sagittal and frontal plane, respectively. Cross sections of anterior spikes (C) JRDI 5ES-232 and (D) JRDI 5ES-260 along the frontal and sagittal plane, respectively. Red arrows indicate axial channel (Scale bars = 10 cm). (TIF) [file pone.0123503.s025.tif]

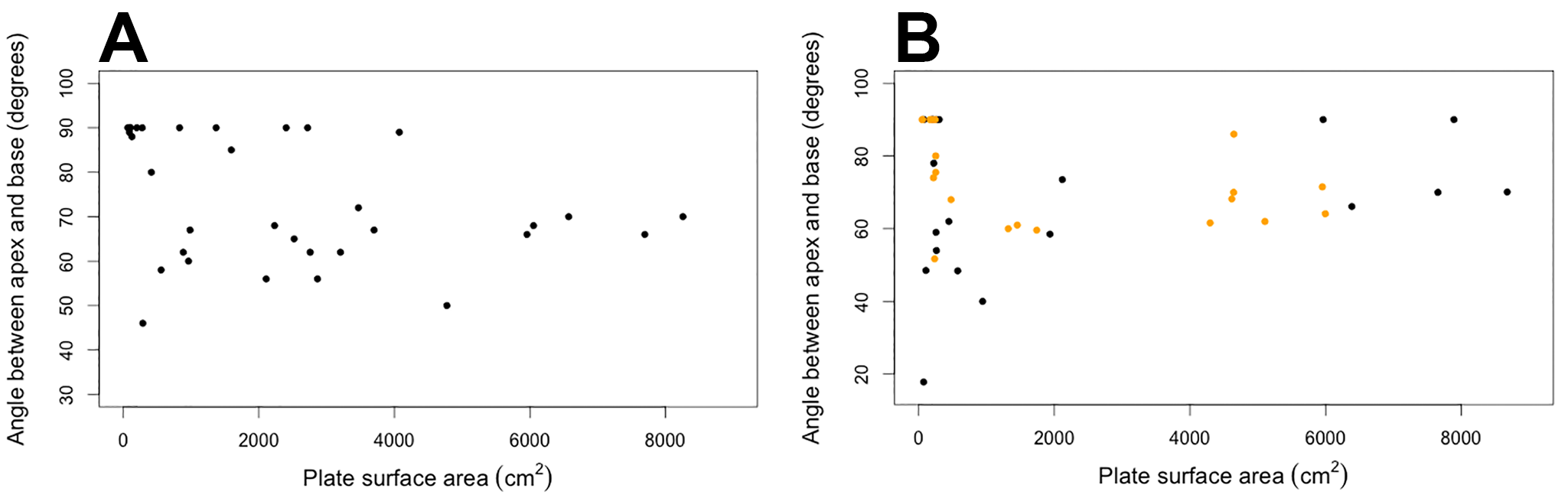

Supplement: S20 Fig — (A) The data from three articulated specimens of Stegosaurus: NHMUK R36730, DMNS 2818, and USNM 4934 (n = 33). (B) The data from the S. mjosi examined in this study. Orange and black points are plates identified to be of the tall and wide morph, respectively (n = 40). (TIF) [file pone.0123503.s026.tif]

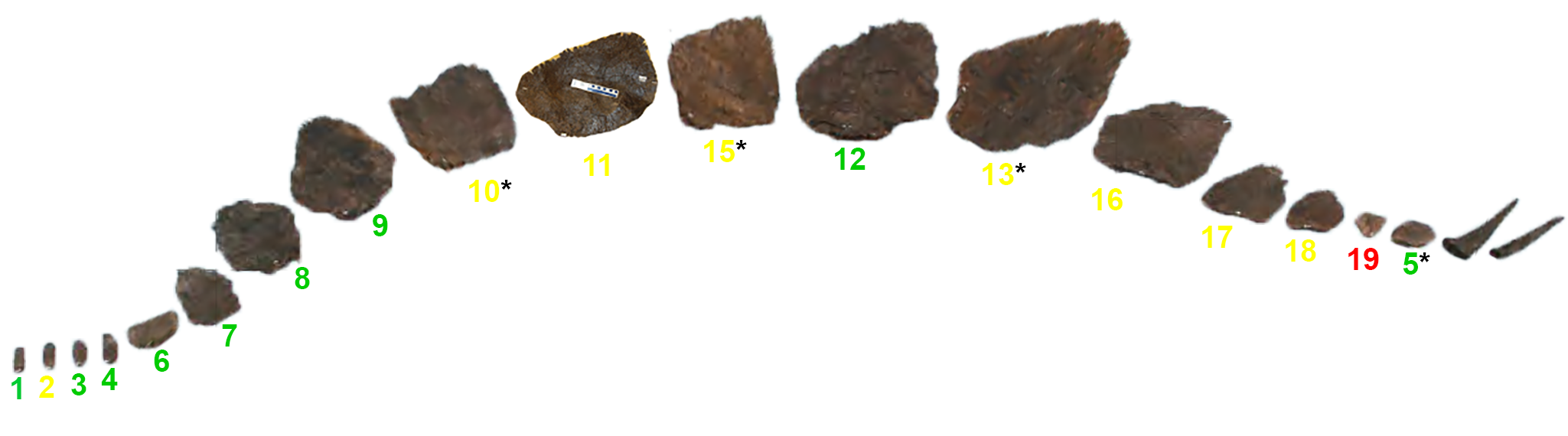

Supplement: S21 Fig — The 18 plates are shown here in correct arrangement from anterior to posterior with the tail spikes shown. The original plate numbers as they were mounted at the Sauriermuseum Aathal are shown below each plate. The color of the number indicates the level of completeness as in S2 Fig The asterisks denote plates that have been rotated by 45° or more and/or flipped in order to properly orient the base of the plate ventrally. Modified from Siber and Möckli [4]. (TIF) [file pone.0123503.s027.tif]
