# Supplementary material for: Evidence for Sexual Dimorphism in the Plated Dinosaur Stegosaurus mjosi (Ornithischia, Stegosauria) from the Morrison Formation (Upper Jurassic) of Western USA
Source: PLoS One. 2015 Apr 22;10(4):e0123503. doi: 10.1371/journal.pone.0123503 (PMC4406738; doi:10.1371/journal.pone.0123503)
Supplement: S1 Table — PCA 1: Loadings of each variable on the first three principal components for a PCA of all fairly complete S. mjosi plates. PC1 explains about 63% of the variation. PC1 and PC2 explain about 83% of the variation. PC1, PC2, and PC3 explain about 98% of the variation (n = 40). PCA 2: Loadings of each variable on the first three principal components for a PCA of all fairly complete S. mjosi plates except the anterior dorsal plate from SMA 0092. PC1 explains about 78% of the variation. PC1 and PC2 explain about 95% of the variation. PC1, PC2, and PC3 explain about 99% of the variation (n = 39). PCA 3: Loadings of each variable for the first three principal components for a PCA of only the most complete S. mjosi plates except the anterior dorsal plate from SMA 0092. PC1 explains about 79% of the variation. PC1 and PC2 explain about 95% of the variation. PC1, PC2, and PC3 explain about 99% of the variation (n = 25). Table corresponds to biplots in Fig 2 and S5 Fig. (DOCX) [file pone.0123503.s029.docx]

|  | | Angle between base and apex | Length of base | Perimeter | ‘Width’ | Distance from centre of the base to apex | Surface area |
| --- | --- | --- | --- | --- | --- | --- | --- |
| PCA 1 | PC1 | 0.01943816 | -0.49496052 | -0.50542783 | -0.49639522 | -0.0933143 | -0.49402751 |
|  | PC2 | 0.58444246 | 0.13454225 | 0.01738103 | -0.11386091 | 0.77520588 | -0.16160083 |
|  | PC3 | 0.8055788 | -0.1061393 | -0.08561 | 0.1390689 | -0.5278847 | 0.1855958 |
| PCA 2 | PC1 | 0.03767213 | -0.44728357 | -0.45787216 | -0.44329133 | -0.43032784 | -0.45517256 |
|  | PC2 | 0.98853924 | 0.01758818 | -0.03633634 | 0.08384578 | -0.07606941 | 0.0913446 |
|  | PC3 | 0.12272422 | -0.25223801 | 0.12062926 | -0.52962941 | 0.78614285 | -0.09074934 |
| PCA 3 | PC1 | 0.09061891 | -0.44128634 | -0.4568545 | -0.44279423 | -0.43148654 | -0.45397267 |
|  | PC2 | 0.98817052 | 0.07474274 | -0.00140655 | 0.05434129 | -0.04352686 | 0.11438094 |
|  | PC3 | 0.092686 | -0.38842871 | 0.21859764 | -0.50675192 | 0.73165727 | -0.02505298 |

Table S1
