# Supplementary material for: Evidence for Sexual Dimorphism in the Plated Dinosaur Stegosaurus mjosi (Ornithischia, Stegosauria) from the Morrison Formation (Upper Jurassic) of Western USA
Source: PLoS One. 2015 Apr 22;10(4):e0123503. doi: 10.1371/journal.pone.0123503 (PMC4406738; doi:10.1371/journal.pone.0123503)
Supplement: S2 Table — Histological stage according to Hayashi et al. [42] and ontogenetic status according to Hayashi et al. [44] listed at the bottom. LAG—Line of arrested growth. (DOCX) [file pone.0123503.s030.docx]

| **Specimen Number** | **JRDI 5ES-256** | | |
| --- | --- | --- | --- |
| **Morph** | **Wide** | | |
|  | **Base** | **Midplate** | **Apex** |
| **Type of bone tissue** | Fibrolamellar:  Reticular channel arrangement | Fibrolamellar;  Laminar/longitudinal channel arrangement | Fibrolamellar;  Laminar/longitudinal channel arrangement |
| **Cyclical or non-cyclical?**  **Number of observable LAGs?** | Azonal;  No LAGs | Zonal;  2 LAGs | Azonal;  No LAGs |
| **Channels** | Many secondary osteons with some simple blood vessels and primary osteons visible | Simple blood vessels twoards the exterior; Many secondary osteons | Many secondary osteons; Some simple blood vessels |
| **Bone types** | Compact bone is mostly secondary and is very thin; Cancellous bone is secondary | Compact bone has a lot of secondary deposition and is very thin; Cancellous bone is secondary | Compact bone has a lot of secondary deposition and is very thin; Cancellous bone is secondary |
| **Classification: Hayashi et al. (2009)** | Histological: Stage 1  Remodeling: Stage 3 | Histological: Stage 3  Remodeling: Stage 3 | Histological: Stage 2  Remodeling: Stage 3 |
| **Classification: Hayashi et al. (2011)** | Structural: Young adult – Old adult  Cortical bone tissue: Old adult  Remodeling: Old adult | | |

Table S2
