# Supplementary material for: Evidence for Sexual Dimorphism in the Plated Dinosaur Stegosaurus mjosi (Ornithischia, Stegosauria) from the Morrison Formation (Upper Jurassic) of Western USA
Source: PLoS One. 2015 Apr 22;10(4):e0123503. doi: 10.1371/journal.pone.0123503 (PMC4406738; doi:10.1371/journal.pone.0123503)
Supplement: S5 Table — Histological stage according to Hayashi et al. [42] and ontogenetic status according to Hayashi et al. [44] listed at the bottom. LAG—Line of arrested growth. (DOCX) [file pone.0123503.s033.docx]

| **Specimen Number** | **JRDI 5ES-525** | | |
| --- | --- | --- | --- |
| **Morph** | **Wide** | | |
|  | **Base** | **Midplate** | **Apex** |
| **Type of bone tissue** | Fibrolamellar;  Reticular channel arrangement | Fibrolamellar;  Laminar/longitudinal channel arrangement | Fibrolamellar;  Laminar/longitudinal channel arrangement |
| **Cyclical or non-cyclical?**  **Number of observable LAGs?** | Azonal;  No LAGs | Too much remodeling to determine | Azonal;  No LAGs |
| **Channels** | Primary osteons with some secondary osteons throughout cortex; Some simple blood vessels | Entirely secondary osteons | Simple blood vessels |
| **Bone types** | Compact bone has some remodeling and is very thin; Cancellous bone is secondary | Compact bone is dense haversian bone and is very thin; Cancellous bone is secondary | Compact bone is primary and is very thin; Cancellous bone is secondary |
| **Classification: Hayashi et al. (2009)** | Histological: Stage 1  Remodeling: Stage 2 | Histological: Unknown  Remodeling: Stage 4 | Histological: Stage 2  Remodeling: Stage 1 |
| **Classification: Hayashi et al. (2011)** | Structural: Young adult – Old adult  Cortical bone tissue: Young adult  Remodeling: Young adult – Old adult | | |

Table S5
