# Supplementary material for: Evidence for Sexual Dimorphism in the Plated Dinosaur Stegosaurus mjosi (Ornithischia, Stegosauria) from the Morrison Formation (Upper Jurassic) of Western USA
Source: PLoS One. 2015 Apr 22;10(4):e0123503. doi: 10.1371/journal.pone.0123503 (PMC4406738; doi:10.1371/journal.pone.0123503)
Supplement: S6 Table — Histological stage according to Hayashi et al. [42] and ontogenetic status according to Hayashi et al. [44] listed at the bottom. LAG—Line of arrested growth. (DOCX) [file pone.0123503.s034.docx]

| **Specimen Number** | **JRDI 5ES-237** | | |
| --- | --- | --- | --- |
| **Morph** | **Tall** | | |
|  | **Base** | **Midplate** | **Apex** |
| **Type of bone tissue** | Fibrolamellar;  Reticular channel arrangement | Fibrolamellar;  Laminar/longitudinal channel arrangement | Fibrolamellar;  Laminar/longitudinal channel arrangement |
| **Cyclical or non-cyclical?**  **Number of observable LAGs?** | Azonal;  No LAGs | Too much remodeling to determine | Zonal;  1 LAG |
| **Channels** | Mostly secondary osteons; A few primary osteons and simple blood vessels | A few simple blood vessels near the exterior; A few primary osteons deeper in the cortex; Most channels are secondary osteons | Simple blood vessels with many secondary osteons |
| **Bone types** | Compact bone is mostly secondary; Cancellous bone is secondary | Compact bone is mostly dense haversian tissue and is very thin; Cancellous bone is secondary | Compact bone has a lot of secondary reconstruction and is very thin; Cancellous bone is secondary |
| **Classification: Hayashi et al. (2009)** | Histological: Stage 1  Remodeling: Stage 4 | Histological: Unknown  Remodeling: Stage 4 | Histological: Stage 3  Remodeling: Stage 3 |
| **Classification: Hayashi et al. (2011)** | Structural: Young adult – Old adult  Cortical bone tissue: Old adult  Remodeling: Old adult | | |

Table S6
