# Supplementary material for: Evidence for Sexual Dimorphism in the Plated Dinosaur Stegosaurus mjosi (Ornithischia, Stegosauria) from the Morrison Formation (Upper Jurassic) of Western USA
Source: PLoS One. 2015 Apr 22;10(4):e0123503. doi: 10.1371/journal.pone.0123503 (PMC4406738; doi:10.1371/journal.pone.0123503)
Supplement: S7 Table — Histological stage according to Hayashi et al. [42] and ontogenetic status according to Hayashi et al. [44] listed at the bottom. LAG—line of arrested growth. EFS—External fundamental system. (DOCX) [file pone.0123503.s035.docx]

| **Specimen Number** | **JRDI 5ES-357** | | |
| --- | --- | --- | --- |
| **Morph** | **Tall** | | |
|  | **Base** | **Midplate** | **Apex** |
| **Type of bone tissue** | Fibrolamellar;  Reticular channel arrangement; Sharpey’s fibers | Fibrolamellar;  Laminar/longitudinal channel arrangement | Fibrolamellar;  Laminar/longitudinal channel arrangement |
| **Cyclical or non-cyclical?**  **Number of observable LAGs?** | Azonal;  No LAGs | Zonal;  3 LAGs | Zonal;  1 interior LAG;  EFS |
| **Channels** | Some simple blood vessels and primary osteons; Many secondary osteons | Simple blood vessels; Many secondary osteons | Mostly simple blood vessels twoards the exterior; Some primary osteons deeper in cortex; Some secondary osteons throughout cortex |
| **Bone types** | Compact bone is mostly secondary and is very thin; Cancellous bone is secondary | Compact bone is mostly secondary and is very thin; Cancellous bone is secondary | Compact bone is mostly primary with some secondary remodeling; Cancellous bone is secondary |
| **Classification: Hayashi et al. (2009)** | Histological: Stage 1  Remodeling: Stage 4 | Histological: Stage 3  Remodeling: Stage 4 | Histological: Stage 4  Remodeling: Stage 3 |
| **Classification: Hayashi et al. (2011)** | Structural: Young adult – Old adult  Cortical bone tissue: Old adult  Remodeling: Old adult | | |

Table S7
