# Supplementary material for: Evidence for Sexual Dimorphism in the Plated Dinosaur Stegosaurus mjosi (Ornithischia, Stegosauria) from the Morrison Formation (Upper Jurassic) of Western USA
Source: PLoS One. 2015 Apr 22;10(4):e0123503. doi: 10.1371/journal.pone.0123503 (PMC4406738; doi:10.1371/journal.pone.0123503)
Supplement: S9 Table — Histological stage according to Hayashi et al. [42] and ontogenetic status according to Hayashi et al. [44] listed at the bottom. LAG—Line of arrested growth. EFS—External fundamental system. (DOCX) [file pone.0123503.s037.docx]

| **Specimen Number** | **JRDI 5ES-552** | | |
| --- | --- | --- | --- |
| **Morph** | **Tall** | | |
|  | **Base** | **Midplate** | **Apex** |
| **Type of bone tissue** | Fibrolamellar;  Reticular channel arrangement; Sharpey’s fibers | Fibrolamellar;  Laminar/longitudinal channel arrangement | Fibrolamellar;  Laminar/longitudinal channel arrangement |
| **Cyclical or non-cyclical?**  **Number of observable LAGs?** | Azonal;  No LAGs | Zonal;  EFS | Zonal;  2 LAGs |
| **Channels** | Mostly primary osteons; Some simple blood vessels twoards the exterior; Secondary osteons present in cortex | Many simple blood vessels twoards the exterior; Some primary osteons deeper in cortex; Secondary osteons throughout cortex | Simple blood vessels twoards the exterior with a few primary osteons deeper in the cortex; Some secondary osteons |
| **Bone types** | Compact bone is mostly primary with some secondary remodeling; Cancellous bone is secondary | Compact bone is mostly primary with some secondary remodeling; Cancellous bone is secondary | Compact bone is mostly primary bone and is very thin; Cancellous bone is mostly secondary |
| **Classification: Hayashi et al. (2009)** | Histological: Stage 4  Remodeling: Stage 3 | Histological: Stage 4  Remodeling: Stage 3 | Histological: Stage 3  Remodeling: Stage 2 |
| **Classification: Hayashi et al. (2011)** | Structural: Young adult – Old adult  Cortical bone tissue: Old adult  Remodeling: Old adult | | |

Table S9
