# Supplementary material for: Evidence for Sexual Dimorphism in the Plated Dinosaur Stegosaurus mjosi (Ornithischia, Stegosauria) from the Morrison Formation (Upper Jurassic) of Western USA
Source: PLoS One. 2015 Apr 22;10(4):e0123503. doi: 10.1371/journal.pone.0123503 (PMC4406738; doi:10.1371/journal.pone.0123503)
Supplement: S11 Table — Histological stage according to Hayashi et al. [42] and ontogenetic status according to Redelstorff & Sander [43] listed at the bottom. No medullary bone was present. LAG—Line of arrested growth. (DOCX) [file pone.0123503.s039.docx]

| **Specimen Number** | **JRDI 5ES-229** | | |
| --- | --- | --- | --- |
|  | **Outer Region** | **Middle Region** | **Inner Region** |
| **Type of bone tissue** | Fibrolamellar;  Laminar/longitudinal channel arrangement | Fibrolamellar;  Laminar/longitudinal channel arrangement | Fibrolamellar;  Laminar/longitudinal channel arrangement |
| **Cyclical or non-cyclical?**  **Number of observable LAGs?** | Zonal;  1 LAG near bone surface | Too much remodeling to determine | Too much remodeling to determine |
| **Channels** | Some simple blood vessels near bone surface; Primary osteons and a few secondary osteons in outermost cortex; Secondary osteons increase in number away from the exterior | Entirely secondary osteons | Entirely secondary osteons |
| **Bone types** | Compact bone has a lot of secondary reconstruction but primary tissue is still visible near bone surface | Compact bone is entirely dense haversian tissue | Compact bone is entirely dense haversian tissue; Cancellous bone is secondary |
| **Classification: Redelstorff & Sander (2009)** | Sexually mature but still growing | | |
| **Classification: Hayashi et. al. (2009)** | Histological: Stage 3  Remodeling: Stage 4 | | |

Table S11
