# Supplementary material for: Evidence for Sexual Dimorphism in the Plated Dinosaur Stegosaurus mjosi (Ornithischia, Stegosauria) from the Morrison Formation (Upper Jurassic) of Western USA
Source: PLoS One. 2015 Apr 22;10(4):e0123503. doi: 10.1371/journal.pone.0123503 (PMC4406738; doi:10.1371/journal.pone.0123503)
Supplement: S12 Table — Histological stage according to Hayashi et al. [42] and ontogenetic status according to Redelstorff & Sander [43] listed at the bottom. No medullary bone was present. LAG—Line of arrested growth. ICL—Inner circumferential layer. (DOCX) [file pone.0123503.s040.docx]

| **Specimen Number** | **JRDI 5ES-501** |
| --- | --- |
| **Type of bone tissue** | Fibrolamellar;  Laminar/longitudinal channel arrangement |
| **Cyclical or non-cyclical?**  **Number of observable LAGs?** | Zonal;  3 LAGs |
| **Channels** | Mostly primary osteons in outer cortex; Primary osteons with a few secondary osteons and large erosional cavities in middle cortex; Dense haversian bone in inner cortex; Large erosional cavities near meduallry cavity; Possible ICL |
| **Bone types** | Compact bone is thick and has secondary deposition up to the middle cortex; No cancellous bone is present |
| **Classification: Redelstorff & Sander (2009)** | Sexually mature but still growing |
| **Classification: Hayashi et. al. (2009)** | Histological: Stage 3  Remodeling: Stage 2 |

Table S12
